# Serum metabolomics of diabetic dogs treated with daily administration of a commercially available lyophilized feces preparation

**DOI:** 10.1007/s11259-026-11181-9

**Published:** 2026-03-27

**Authors:** Jose D. J. Ruiz Romero, Patrick Barko, Jan S. Suchodolski, David A. Williams, Holly H. Ganz, Arnon Gal

**Affiliations:** 1 Department of Veterinary Clinical Medicine, University of Illinois Urbana- Champaign, Urbana, IL 61802 USA; 2 Gastrointestinal Laboratory, Department of Small Animal Clinical Sciences, Texas A&M University, College Station, TX 77845 USA; 3 AnimalBiome, Oakland, CA 94609 USA; 4 Pacific Care Pet Emergency and Specialty, Irvine, CA 92614 USA

**Keywords:** Canine diabetes mellitus, Gut microbiome modulation, Insulin sensitivity, Metabolite profiling, Randomized controlled trial

## Abstract

Enteric microbial dysbiosis can contribute to the pathobiology of canine diabetes mellitus (DM) by interfering with host metabolism, promoting inflammation, and altering incretin hormone responses. Enriching and diversifying an unbalanced gastrointestinal microbiome with microbiome-based interventions such as lyophilized fecal preparations (LFP) can help improve gut function and glycemic control of diabetic dogs. This study aimed to determine the differences in the serum metabolomes, levels of interstitial glucose (IG), and 24-hour water intake of diabetic dogs treated with daily administration of LFP or placebo. A prospective, randomized, double-masked, placebo-controlled clinical trial was conducted using six DM dogs in the LFP group and six DM dogs in the placebo group. Dogs had physical examinations and 24-hour water intake assessments at admission, 2, 4, 6, and 8 weeks, with continuous IG monitoring performed throughout the study. Serum samples were collected longitudinally at baseline and during follow-up for untargeted metabolomic analysis. The marginal mean of IG after 8 weeks was lower in the LFP group (286 ± 22 mg/dL vs. 309 ± 22 mg/dL; *p* = 0.468), with the LFP group showing lower marginal mean IG at each time point except week five. Marginal mean log 24-hour water intake was significantly lower in the LFP group compared to placebo (mean difference − 0.24 ± 0.10, *p* = 0.024). Treatment groups significantly differed in metabolites with important roles in carbohydrate, protein, and lipid metabolism. Overall, our results show that LFP induced measurable metabolic alterations, with patterns generally consistent with a more favorable metabolic profile, but only minimal improvement in glycemic control.

**Supplementary Information:**

The online version contains supplementary material available at 10.1007/s11259-026-11181-9.

## Introduction

Diabetes mellitus (DM) is a complex disease characterized primarily by hyperglycemia resulting from inadequate insulin secretion, reduced insulin sensitivity, or both. Given its multifactorial nature, there is significant interest in better understanding the pathogenesis of DM and exploring novel therapeutic interventions to enhance its management. One area gaining attention is the role of the gastrointestinal (GI) microbiome in influencing metabolic health and disease.

Disruption of the GI microbiome, or dysbiosis, has been increasingly implicated in the pathophysiology of DM (Psichas et al. 2015). Dysbiosis in individuals with diabetes had been associated with altered abundances of gut bacteria that ferment dietary fiber into metabolites, such as short-chain fatty acids (SCFAs), which are known to influence insulin sensitivity (Lau and Vaziri 2019; Lin et al. 2012; Tolhurst et al. 2012). Specifically, reductions in SCFA-producing bacteria, including butyrate producers, have been observed in diabetic states. Butyrate itself is notable for promoting the secretion of glucagon-like peptide-1 (GLP-1), thereby stimulating insulin release from pancreatic beta cells. Additionally, gut dysbiosis and diabetes might induce chronic systemic inflammation by compromising intestinal barrier integrity, further exacerbating insulin resistance (Crakes et al. 2021; Lau and Vaziri 2019; Tanase et al. 2020).

Fecal microbiota transplantation (FMT), the transfer of fecal material from a healthy donor into the GI tract of a recipient, has become a promising method for microbiome modulation. In a key 2012 double-blind, randomized controlled trial, weekly FMT from lean donors to human patients with metabolic syndrome significantly improved insulin sensitivity and increased gut populations of butyrate-producing bacteria (Vrieze et al. 2012). Considering this potential, exploring FMT as a treatment option for managing DM in veterinary medicine, especially in diabetic dogs, could provide valuable clinical insights and additional treatment options alongside dietary changes and insulin therapy. Although FMT has traditionally been described as the transfer of fresh or minimally processed donor feces via endoscopic or enema-based routes, contemporary definitions emphasize the process of microbiota transfer rather than the delivery method or source of donor material. In human medicine, FMT is now commonly administered orally using encapsulated stool-derived preparations, and randomized controlled trials have demonstrated that oral capsule–based FMT is non-inferior to colonoscopic delivery for the prevention of recurrent *Clostridioides difficile* infection (Kao et al. 2017). In addition, repeated oral dosing regimens have been used in human studies aimed at sustained microbiome modulation beyond acute infectious indications, supporting the biological plausibility of serial administration approaches.

The intervention used in the present study, a commercially prepared, orally administered lyophilized fecal preparation (LFP) derived from screened healthy canine donors, represents a standardized, capsule-based FMT strategy (Youngster et al. 2014, 2016). While this formulation differs operationally from some classical FMT protocols (e.g., fresh stool, endoscopic delivery), it remains mechanistically aligned with FMT principles in that it delivers donor-derived microbial communities and biologically active fecal components intended to modulate the recipient gut ecosystem (Mullish et al. 2018). Importantly, commercial sourcing does not alter the fundamental nature of FMT as a therapeutic process but rather reflects increasing efforts toward standardization, safety screening, and clinical scalability.

Metabolomics, the comprehensive profiling of small-molecule metabolites within biological samples, offers a powerful method to explore metabolic changes related to DM and to monitor responses to treatments. For example, an untargeted metabolomic analysis identified unique metabolite profiles that distinguish diabetic dogs from healthy ones, demonstrating the potential of metabolomics in characterizing disease states and therapeutic effects (O’Kell et al. 2017). Although studies like these in canine diabetes remain limited, combining metabolomic data with microbiome insights could significantly enhance understanding of DM development and inform the development of targeted treatments.

Our overarching hypothesis is that enteric microbial dysbiosis contributes to the development of canine DM by disrupting host metabolism, promoting systemic inflammation, and altering incretin hormone responses. Given that LFP has the potential to restore beneficial gut-derived metabolites, our objectives were to determine whether an 8-week regimen of daily administration of a commercially available LFP influences the serum metabolome in diabetic dogs. As a control measure, we also assessed whether LFP would lower interstitial glucose levels and 24-hour water intake relative to those of diabetic dogs in a placebo control group.

## Methods (detailed methods are available in Supplementary File 1)

### Study design

A prospective, randomized, placebo-controlled, double-blinded clinical trial was conducted at the University of Illinois from September 2021 to June 2022 (IACUC#19235). Twelve diabetic dogs recruited from public sources and local referral clinics were randomly assigned to receive either daily administration of a commercially available LFP or a placebo. Eligible dogs had confirmed stable diabetes mellitus (DM), received insulin therapy for at least 14 days, maintained stable body weight (± 5% baseline), showed no obvious diabetic clinical signs, and had no prior exposure to steroids, antibiotics, or probiotics for 14 days. Clinical evaluations were performed at baseline and at weeks 2, 4, 6, and 8, including a physical examination, complete blood count (CBC), serum biochemical profile, and urinalysis. The CBC, serum biochemical profiles, and urinalysis were part of routine clinical monitoring to ensure patient stability and detect adverse events, rather than as predefined efficacy endpoints. These data were not used for hypothesis testing. All dogs wore flash glucose monitors with readings uploaded to a secure account every 6 h.

### Insulin treatment and LFP administration

All dogs transitioned to Toujeo glargine insulin (every 12 h), with dosing matched to pre-enrollment levels. Dogs weighing ≤ 15 kg received Toujeo SoloStar, while those > 15 kg received Toujeo Max SoloStar. Owners adjusted insulin daily using a standardized sliding-scale based on postprandial interstitial glucose measured 60 min after feeding. LFP-treated dogs received 1 g daily lyophilized fecal material (0.035–0.167 g/kg) encapsulated for oral delivery, sourced from AnimalBiome Inc. (Oakland, CA). Identically sized placebo capsules contained cornstarch (1 g; 0.053–0.192 g/kg), which is not a prebiotic at administered doses. A veterinary pharmacist, blind to all other study activities, managed randomization and capsule distribution.

### Metabolomic analyses

Untargeted metabolomics was performed at the University of Illinois Urbana-Champaign’s Roy J. Carver Biotechnology Center on serum samples using LC-MS (Dionex Ultimate 3000 UHPLC with Q-Exactive MS) and GC-MS (Agilent 7890 gas chromatograph). LC-MS samples were analyzed via reversed-phase chromatography with detection in positive and negative ionization modes, using MS-DIAL software for peak detection and identification against in-house and NIST libraries. GC-MS employed a ZB-5MS capillary column with electron ionization detection. Known metabolites matched to chemical standards were classified as MSI level 1. Lipid indices included SCD1_16 (16:1/16:0), SCD1_18 (18:1/18:0), and de novo lipogenesis (DNL) ratios calculated from GC-MS intensities. Long-chain acylcarnitines were derived from LC-MS data.

### Statistical analyses

Statistical analyses were performed using SAS v9.4 (SAS Institute Inc., Cary, NC) and R (Version 2023.06.2 + 561). Normality was assessed using histograms, q–q plots, and Shapiro–Wilk tests; descriptive statistics are reported as mean (± SD) or median (min, max), as appropriate. Linear mixed models analyzed interstitial glucose and log-transformed 24-hour water intake, accounting for repeated measures within dogs (fixed effects: treatment, week, and treatment × week; random effect: dog). Pairwise comparisons were performed using Fisher’s least significant difference with Tukey adjustment. Ordinal BCS, and non-normally distributed age and body weight were evaluated using nonparametric methods.

Metabolomic features were filtered and imputed, then median-scaled and natural-log transformed. Unsupervised analyses included PCA and hierarchical clustering. Serum metabolite abundance data were analyzed using linear mixed-effects models to account for repeated measurements within individual dogs across the study period. Metabolite abundances were median-centered and log-transformed prior to analysis. For each metabolite, models included fixed effects for treatment group (phenotype), time (treated as a categorical variable; weeks 0, 2, 4, 6, and 8), and the treatment × time interaction, with a random intercept for subject to account for within-dog correlation: metabolite ~ phenotype + time + phenotype × time + (1 | subject). This modeling approach incorporated baseline and all follow-up metabolomic samples, allowing assessment of overall treatment effects while accounting for temporal variation and inter-individual heterogeneity. Pairwise contrasts of estimated marginal means were performed where appropriate, and false discovery rates across metabolites were controlled using q-values (q < 0.2). Post-hoc contrasts were estimated using emmeans. Metabolite set enrichment analysis evaluated overrepresentation of metabolic sub-pathways using fgsea, ranking metabolites by a signed composite statistic incorporating effect size (log2–fold change) and significance. Lipid indices and long-chain acylcarnitines were derived from GC–MS/LC–MS data, natural-log transformed (offset 0.001), and analyzed using mixed-effects models with AR(1) repeated measures. Additional details, including sample size justification and full metabolomics preprocessing and enrichment workflows, are provided in Supplementary File 1.

## Results

### Demographic characteristics and clinical outcomes

No differences in age (*p* = 0.229), body weight (*p* = 0.128), or body condition score (*p* = 0.931) were detected between placebo and LFP-treated diabetic dogs (Supplementary File 1). LFP-treated dogs demonstrated lower log 24-hour water intake compared to placebo (1.64 ± 0.07 vs. 1.87 ± 0.07; *p* = 0.024) (Supplementary Fig. 1). In LFP-treated dogs, log 24-hour water intake declined over the study period relative to baseline (*p* = 0.01). Interstitial glucose levels were lower in the LFP group (286.13 ± 21.78 mg/dL) versus placebo (308.49 ± 21.78 mg/dL; *p* = 0.468) (Supplementary Fig. 2). Both groups exhibited increased glucose levels during weeks 1–2 compared with subsequent time points (*p* < 0.001). Routine clinical laboratory parameters, including CBC, serum biochemistry, and urinalysis, did not reveal treatment-associated abnormalities or clinically meaningful indicators of systemic inflammation. Descriptive summaries are provided in Supplementary Files 3–6.

### Metabolomic analyses

Metabolomic analyses incorporated baseline and longitudinal serum samples collected at weeks 0, 2, 4, 6, and 8, and treatment effects were evaluated using repeated-measures mixed-effects models. Thus, reported metabolite differences reflect treatment-associated effects estimated using longitudinal data across the study period.

**Fig. 1:**
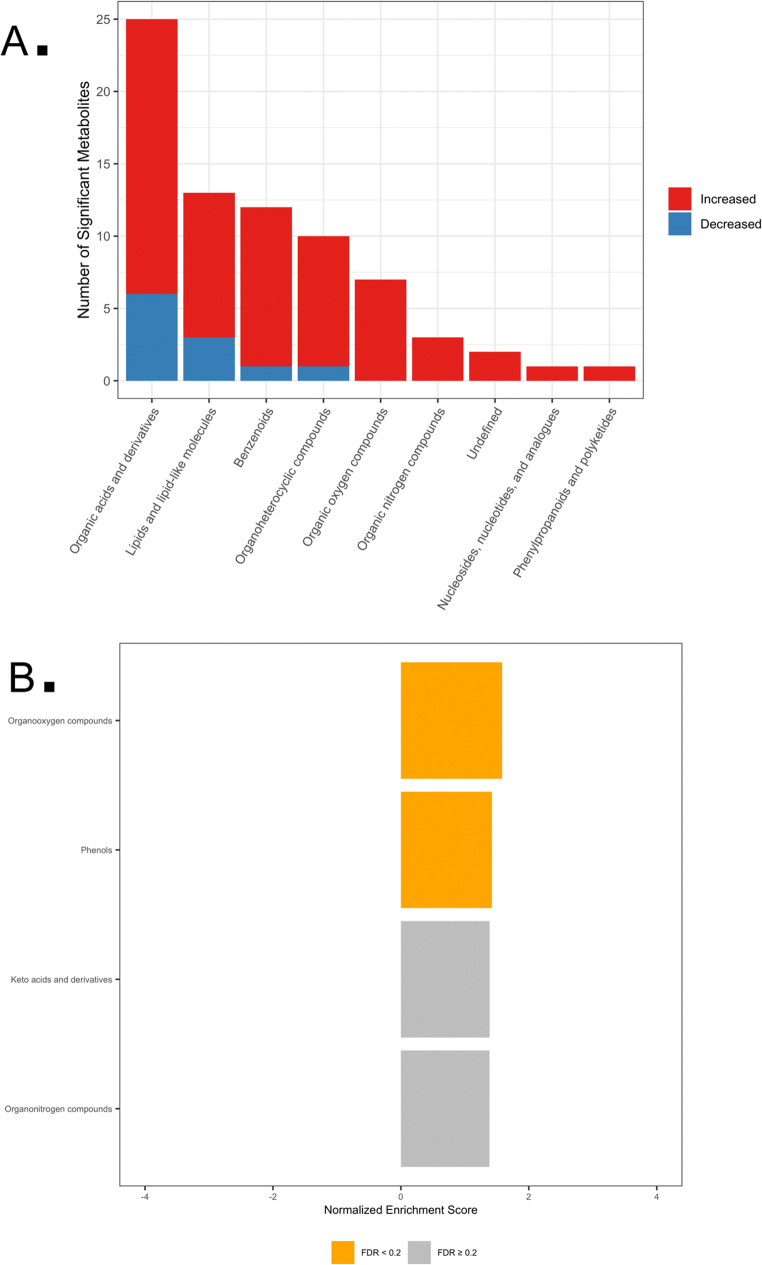
GC-MS metabolic pathway analysis. **A** Counts of significantly variable metabolites by metabolic super-pathway. Red and blue bars represent increased and decreased metabolites, respectively, in placebo versus LFP groups. **B** Metabolite Set Enrichment Analysis (MSEA) of sub-pathways showing enriched pathways among metabolites with low relative abundance (negative NES). Bars annotated with FDR-adjusted *p*-values (*q*-values)

GC-MS analysis identified 73 serum metabolites across eight super-pathways, with 16 metabolites showing significant differences between groups (q < 0.2). Eight metabolites were higher and eight lower in placebo relative to LFP, with sucrose, citric acid, and unsaturated fatty acids (11-octadecenoic acid, 9,12-octadecadienoic acid) showing the largest effect sizes (Tables 1 and 2 in Supplementary File 2). Metabolite set enrichment analysis revealed lower fatty acid enrichment in placebo-treated dogs (NES − 1.538; *p* = 0.011; q = 0.059) (Fig. 1).

**Fig. 2:**
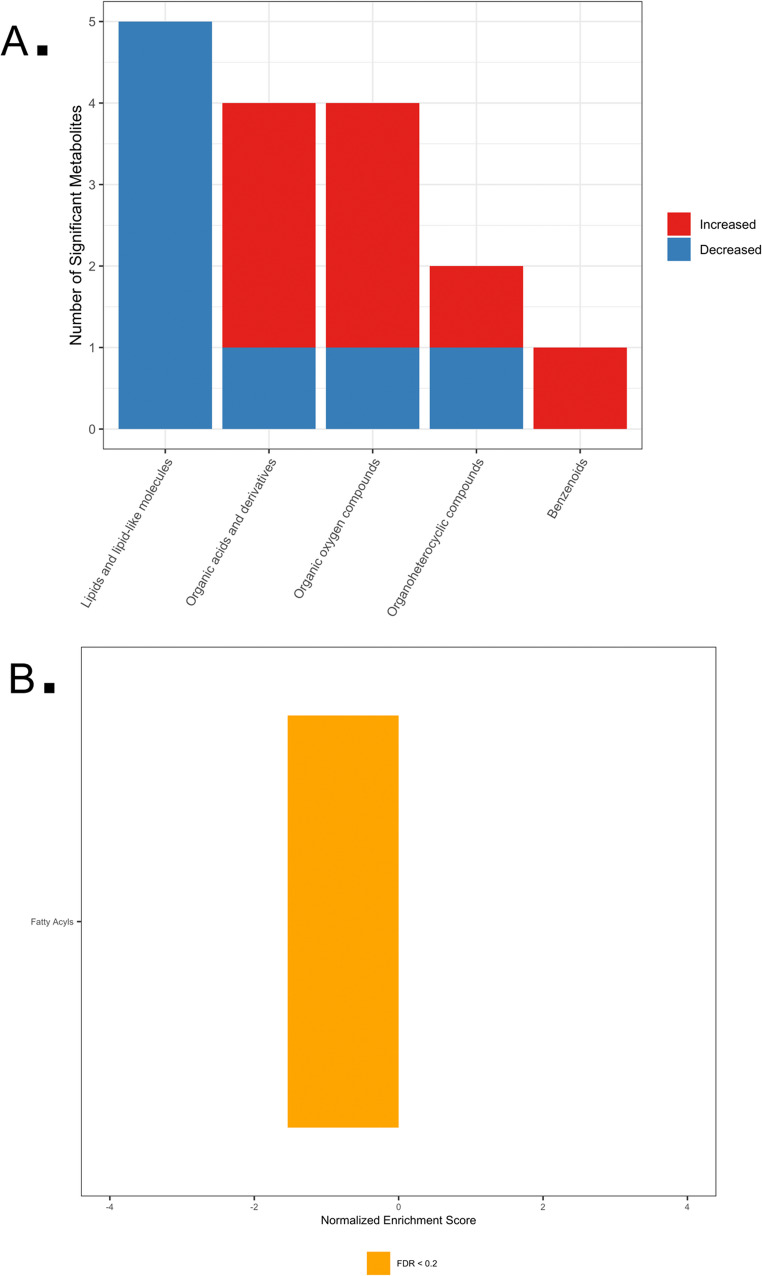
LC-MS metabolic pathway analysis. **A** Counts of significantly variable metabolites by metabolic super-pathway. Red and blue bars represent increased and decreased metabolites, respectively, in placebo versus LFP groups. **B** Metabolite Set Enrichment Analysis (MSEA) showing sub-pathways with high (positive NES) and low (negative NES) relative abundance. Bars annotated with FDR-adjusted *p*-values (*q*-values)

LC-MS identified 392 serum metabolites across 11 super-pathways, with 74 metabolites that differed between groups (q < 0.2). Sixty-four metabolites were higher and ten lower in placebo relative to LFP. Key affected metabolites included linoleic acid, stearoyl-L-carnitine, lysine, homoarginine, allantoin, and daidzein 4’-sulfate (Table 3, and 4 in Supplementary File 2). MSEA highlighted enrichment of organooxygen compounds (NES 1.583; *p* = 0.014) and phenols (NES 1.423; *p* = 0.013) in placebo-treated dogs (Fig. 2).

### Lipid metabolism

LFP-treated dogs exhibited higher stearoyl-CoA desaturase (SCD1_16; *p* = 0.033) and lower de novo lipogenesis (DNL; *p* = 0.018) compared to placebo (Table 1). Long-chain acylcarnitines were lower in LFP dogs for stearoyl-carnitine C18 (*p* = 0.024), and palmitoyl-carnitine C16 was borderline lower (*p* = 0.121). This pattern suggests enhanced desaturation capacity and reduced lipogenic activity, accompanied by decreased markers of incomplete fatty acid oxidation in LFP-treated dogs.

**Table 1:** Least-squares means (LSM ± SE) from mixed-effects models for GC–MS–derived lipid indices and LC–MS–measured long-chain acylcarnitines in LFP-treated and placebo groups

Variable	Placebo		LFP		*P* value
	LSM	SE	LSM	SE	
Log SCD1_16	-4.709	0.576	-2.699	0.576	0.033
Log SCD1_18	-0.021	0.304	0.138	0.304	0.718
Log DNL	0.587	0.0799	0.267	0.080	0.018
Log DNL_alt	0.645	0.0989	0.424	0.099	0.146
Log palmitoyl_carnitine_C16	16.565	0.161	16.179	0.161	0.121
Log stearoyl_carnitine_C18	16.970	0.184	16.280	0.184	0.024

*DNL* De novo lipogenesis, *LFP *Lyophilized feces preparation, *LSM *Least squares means, *SCD1 *Stearoyl-CoA desaturase 1, *SE *Standard error

## Discussion

Untargeted serum metabolomic profiling revealed differences in groups of metabolites related to carbohydrate, protein, and lipid metabolism between LFP-insulin-treated and placebo-insulin-treated dogs. These metabolomic differences suggest that eight weeks of daily administration of LFP have some effects on the metabolic state of insulin-treated diabetic dogs. While LFP-treated dogs demonstrated minimal clinical benefits, including lower 24-hour water intake compared to placebo (log-transformed water intake 1.64 ± 0.07 vs. 1.87 ± 0.07; *p* = 0.024), the mean IG concentrations did not differ between groups. Since polydipsia in diabetes is mechanistically linked to renal tubular urinary glucose load, which is mediated mainly through sodium-glucose co-transporters in the renal proximal tubule and downstream juxtaglomerular apparatus feedback mechanisms that regulate the glomerular filtration rate (Gal and Burchell 2023; Vallon and Thomson 2012), the lower water intake could suggest some degree of improvement in glycemic regulation. As we further explain below, the daily administration of LFP was associated with metabolomic changes in metabolic pathways that could be considered “favorable”. However, the lack of a significant reduction in IG in the LFP-treated group relative to the placebo group, as well as the small sample size, does not provide evidence of clinical efficacy.

Accumulating evidence suggests that dysbiosis exacerbates metabolic dysfunction through multiple mechanisms: promoting insulin resistance, impairing immune homeostasis, altering incretin hormone release, reducing intestinal gluconeogenesis, increasing gut permeability, and enabling overgrowth of proinflammatory bacteria (Pedersen et al. 2016). Conversely, enriching microbial diversity through FMT-based microbiome modulation strategies, including LFP, could counter these abnormalities by restoring a eubiotic microbiome, thereby improving hepatic and peripheral insulin sensitivity and reducing systemic inflammation. Human studies have demonstrated that transferring a healthy microbiota via FMT-based approaches improves insulin sensitivity in metabolic syndrome (Vrieze et al. 2012), which could be particularly relevant to diabetic patients (and dogs) who often have absolute or functional insulin deficiency, persistent hyperglycemia, enhanced lipolysis, and a catabolic state (Abu-Lebdeh and Nair 1996; Gal and Odunayo 2023; Morigny et al. 2016). Our findings align with this concept and provide preliminary evidence that modulating the gut microbiome through daily administration of LFP can benefit the perturbed metabolic state in diabetic dogs.

Metabolomic analysis identified several serum metabolites that differed between the LFP and placebo groups, providing physiologic insight into the metabolic perturbations in diabetic dogs. Circulating free fatty acids were generally lower in placebo-treated dogs. The LFP group showed a higher SCD1 (Δ9-desaturase) signature [i.e., significantly greater log (16:1/16:0)] and lower de novo lipogenesis [DNL; i.e., lower log (16:0/18:2)] relative to placebo, alongside lower long-chain acylcarnitines (notably C18). Because product-to-substrate ratios of 16:1/16:0 and 18:1/18:0 are widely used in vivo as proxies of SCD1 activity, an increase in these indices can be interpreted as greater desaturation capacity rather than generalized lipogenesis. In contrast, the 16:0/18:2 (palmitate/linoleate) ratio is a standard blood-lipid marker of carbohydrate-driven hepatic DNL. Falling DNL values argue against increased net fatty-acid synthesis (Chong et al. 2008; Hodson and Fielding 2013; Saponaro et al. 2015). Furthermore, increased circulating serum long-chain acylcarnitines typically reflect incomplete mitochondrial β-oxidation and correlate with insulin resistance; their lower levels in the LFP group are therefore consistent with improved oxidative handling (Schooneman et al. 2013). Taken together, the pattern of higher desaturation without higher DNL and lower LC-acylcarnitine is consistent with a metabolic profile associated with better insulin responsiveness in LFP recipients. This interpretation aligns with randomized clinical trials in humans, which show that lean-donor FMT improves peripheral insulin sensitivity in patients with metabolic syndrome. In essence, LFP-treated dogs exhibited a metabolic profile more closely aligned with biochemical patterns associated with improved insulin action, relative to placebo-treated dogs, as reflected by the SCD1and DNL indices, and lower long-chain acylcarnitine levels.

We also observed that glycerate, allantoin, and stearoyl-carnitine were present at lower concentrations in LFP-treated dogs compared to placebo controls. Each of these metabolites is associated with hyperglycemia or insulin resistance. Glycerate can accumulate when persistent hyperglycemia suppresses efficient glycolysis, serving as a marker of perturbed glucose metabolism. Allantoin is the oxidation product of uric acid; in most mammals (including dogs, which express urate oxidase), excess uric acid from hyperglycemia-induced purine turnover is converted to allantoin (Cicero et al. 2023; Haythorne et al. 2022). Increased serum allantoin, therefore, can reflect heightened oxidative stress and inflammation secondary to poor glycemic control (Rafiullah and Siddiqui 2022). Stearoyl-carnitine (stearoyl-L-carnitine), a long-chain acylcarnitine, has been implicated in β-cell dysfunction; prior studies in diabetic mice found accumulation of acylcarnitine in pancreatic islets, which was linked to impaired insulin synthesis and secretion (Aichler et al. 2017). Thus, higher levels of glycerate, allantoin, and stearoyl-carnitine can be viewed as supportive of under-regulated diabetes with insulin resistance and oxidative stress. The fact that all three were much lower in the LFP group provides further support for the assertion that daily administration of LFP was associated with attenuation of metabolic perturbations linked to poor glycemic regulation, relative to placebo-treated dogs. In other words, the daily administration of LFP was associated with fewer metabolic perturbations than in diabetic dogs receiving a placebo.

Markers of inflammation and immune activation were also favorably altered by LFP. The LFP-treated dogs had lower serum levels of kynurenine, citric acid, and N-acetylneuraminate (sialic acid) compared to the placebo dogs. Chronic, low-grade inflammation is a well-recognized feature of diabetes that can aggravate insulin resistance (de Luca and Olefsky 2008). Kynurenine is a product of tryptophan catabolism via the indoleamine 2,3-dioxygenase (IDO) pathway, and increased kynurenine levels have been observed in poorly regulated diabetic humans (Oxenkrug 2013). Modulation of the kynurenine pathway has pharmacologic appeal: in rodent experimental studies and human clinical trials, reducing kynurenine accumulation (by inhibiting downstream enzymes) enhanced glucose-stimulated insulin release and improved glucose metabolism (Hoffman et al. 2021; Koziel and Urbanska 2023). The lower kynurenine levels in LFP-treated dogs could reflect a dampening of this inflammation-linked pathway, thereby potentially contributing to improved insulin secretion or sensitivity. Citric acid is a TCA-cycle intermediate that tends to accumulate in the serum of systemically inflamed insulin-resistant rodents, possibly reflecting mitochondrial dysfunction or cell injury (Satapati et al. 2012). The lower serum citrate in LFP-treated dogs, therefore, aligns with reduced metabolic stress and inflammation. Meanwhile, sialic acid (N-acetylneuraminic acid) is recognized as a biomarker of inflammation and has been linked to macrophage activation and vascular dysfunction in metabolic disease (Hou et al. 2019). In our study, the LFP group’s lower levels of citric and sialic acids compared to placebo are consistent with a lower systemic inflammatory state. Collectively, these differences in metabolites linked to inflammation could support the interpretation that LFP-treated dogs had less inflammation, which, in turn, could facilitate more favorable insulin–sensitivity–related metabolic signaling.

LFP-treated dogs showed higher circulating levels of certain essential nutrients, specifically linoleic acid, lysine, and homoarginine, and lower sucrose levels. These findings could reflect improvements in intestinal absorption and barrier integrity. Gut dysbiosis in diabetes can lead to an overgrowth of pathogenic Gram-negative bacteria, increased luminal lipopolysaccharide (LPS), and greater intestinal permeability (Tanase et al. 2020). Linoleic acid is an essential dietary fatty acid, lysine is an essential amino acid, and homoarginine is a lysine derivative that serves as a cardiovascular and metabolic biomarker (Davids et al. 2012; van Rooijen et al. 2014; Vastolo et al. 2022). The higher serum levels of linoleic acid, lysine, and homoarginine in LFP dogs could indicate more effective absorption or conservation of these nutrients. In contrast, in placebo dogs with dysbiosis, lower serum levels of linoleic acid, lysine, and homoarginine could result from malabsorption or from greater utilization. Conversely, sucrose is usually broken down by intestinal disaccharidases before its monosaccharides are absorbed (Bona et al. 2022). Increased gut permeability might allow some intact sucrose to cross the gut epithelium, possibly explaining the elevated circulating sucrose in placebo dogs. The lower sucrose levels and higher essential nutrient levels in the LFP group suggest a healthier gut lining and improved nutrient uptake.

This study has several important limitations that warrant discussion. First, while six dogs per group is a small sample in absolute terms, comparable sample sizes have successfully identified significant metabolomic differences in canine research. Notably, O’Kell et al. (2017) conducted an untargeted metabolomic study of diabetic dogs with an identical sample size (*n* = 12 total) and detected meaningful metabolite differences between diabetic and healthy controls. Despite the modest cohort size, we identified 74 metabolites that differed between groups by LC-MS and 16 by GC-MS, with appropriate false discovery rate corrections, demonstrating that robust and biologically relevant effects can be detected at this sample size. Nevertheless, larger studies can reveal additional metabolites or stronger effect sizes currently obscured by limited statistical power.

Second, diet and feeding regimens were not uniform across all dogs, and diet types were not recorded. While this represents a potential confounder, it also reflects real-world clinical conditions. In practice, if daily administration of LFP is to be therapeutically useful in managing canine diabetes, it must demonstrate efficacy across diverse dietary backgrounds rather than exclusively under highly controlled laboratory conditions. The ability to observe significant metabolomic effects despite non-standardized diets actually strengthens the argument for the robustness and clinical relevance of LFP-induced changes. Nonetheless, studies that standardize diet or employ statistical adjustment for nutritional variables would help isolate LFP-specific effects from diet-driven metabolic changes.

Third, a key consideration is that the intervention employed in this study was an orally administered LFP rather than a single-administration, endoscopically delivered FMT. While oral capsule–based stool-derived microbiome therapies, including LFP, have been shown to be clinically effective and non-inferior to colonoscopic delivery in human studies, formulation, processing (e.g., lyophilization), dosing frequency, and delivery route may influence microbial viability, engraftment, and downstream biological effects. Consequently, the metabolomic changes observed here may reflect not only microbiota transfer per se but also the specific pharmacologic and kinetic characteristics of repeated oral administration of stool-derived material. These distinctions should be considered when extrapolating findings across different FMT modalities.

Fourth, we did not confirm the viability of the transplanted microbes in the lyophilized product or perform metagenomic analysis to characterize microbial populations. However, this limitation warrants nuance. Emerging evidence suggests that intact, viable bacterial cells are not always necessary for FMT efficacy; studies have demonstrated biological activity from bacterial cellular components derived from dead or non-viable bacteria. For instance, Ott et al. (2017) demonstrated that sterile fecal filtrate lacking living organisms but retaining bacterial cellular components was effective in treating *Clostridium difficile* infection in humans, suggesting that bioactive bacterial cellular material, rather than viable microorganisms, could have mediated the LFP metabolomic effects observed in our study. Nevertheless, studies characterizing the microbiota of donor and recipient dogs throughout treatment would help elucidate the specific microbial and metabolomic mechanisms underlying any observed benefits.

Also, there was variability in LFP donor material due to logistical constraints: some dogs received LFP from a single donor, while others received a pooled multi-donor product. The optimal donor composition for daily administration of LFP in diabetes remains unknown, and we did not characterize the donor fecal microbiota or metabolite profiles. While we acknowledge it as a methodological limitation, emerging evidence suggests that this variability could not substantially compromise therapeutic efficacy. A systematic review and meta-analysis of 14 controlled studies in ulcerative colitis demonstrated that pooled multi-donor FMT was significantly superior to single-donor FMT in achieving treatment response, with pooling associated with enhanced microbial diversity and reduced batch-to-batch variability (Levast et al. 2023). Pooling different donors enriches the fecal product and increases the repertoire of beneficial bacteria and functionalities, potentially improving clinical outcomes by increasing the probability of donor-recipient compatibility (Kazerouni and Wein 2017; Levast et al. 2023). Consequently, the heterogeneity in donor composition observed in our study, rather than being a limitation, could have enhanced microbial diversity in recipients receiving pooled-donor LFP.

Fifth, although metabolomic data were analyzed using longitudinal mixed-effects models incorporating baseline and follow-up samples, this study was not powered to fully characterize detailed time-course dynamics for individual metabolites. While principal component analysis showed considerable overlap between groups, we nevertheless identified 74 significantly different metabolites at false discovery rate correction (q < 0.2), indicating that, despite global similarity, specific metabolic pathways do diverge between treatment groups. Importantly, examining discrete metabolite differences through rigorous statistical correction provides complementary information to global clustering patterns. The metabolite-specific discrepancies, particularly in markers of inflammation, glycemic control, and lipid metabolism, suggest that, albeit modest in magnitude, LFP successfully mitigated some of the metabolic perturbations associated with diabetes. Detailed temporal tracking of individual metabolites and treatment-by-time interactions remains an important avenue for further investigation to understand the kinetics of metabolomic response to daily administration of LFP.

Sixth, this study did not include comprehensive, predefined assessments of inflammatory cytokines or incretin hormone responses. Although CBC data did not suggest clinically relevant systemic inflammation, these measures are nonspecific. Similarly, serum CRP and GLP-1 concentrations were available only in a subset of dogs due to technical limitations and lack of remaining serum, precluding meaningful statistical analysis. As such, conclusions regarding inflammatory or incretin-mediated mechanisms are based primarily on metabolomic signatures rather than direct hormone or cytokine quantification.

Lastly, metabolomic associations do not establish causation. The metabolic changes observed could reflect consequences of improved glycemic regulation rather than direct effects of daily administration of LFP, or vice versa. We present a plausible framework for how LFP might improve metabolic health in diabetic dogs, but our findings remain correlative and hypothesis-generating. Larger, adequately powered studies are essential to confirm LFP efficacy. Investigations should incorporate standardized or nutritionally characterized diets to segregate diet-driven from LFP-driven metabolic effects. Characterizing donor and recipient microbiota throughout the study, including metagenomic profiling and assessment of bacterial viability, would clarify whether engraftment of live organisms or transfer of bacterial cellular components drives the metabolomic changes. Exploring variations in LFP/FMT-derived preparation protocols (single versus multi-donor, fresh versus lyophilized, dosing frequency) would identify optimal strategies for microbiome modulation. Finally, incorporating direct measures of insulin sensitivity, inflammatory cytokine levels, incretin hormone levels, and longitudinal metabolomic profiling would elucidate the temporal sequence and causal relationships among microbial, metabolomic, and clinical changes in diabetic dogs treated with daily LFP administration.

## Supplementary Information

Supplementary Material 1.

Supplementary Material 2.

Supplementary Material 3.

Supplementary Material 4.

Supplementary Material 5.

Supplementary Material 6.

Supplementary Material 7.

Supplementary Material 8.

## Data Availability

Data presented in this study are available on request from the corresponding author.

## Abbreviations

AR(1): Autoregressive correlation structure of order 1
BCS: Body condition score
DM: Diabetes mellitus
DNL: De novo lipogenesis
GC-MS: Gas chromatography–mass spectrometry
GI: Gastrointestinal
GLP-1: Glucagon-like peptide-1
IACUC: Institutional animal care and use committee
IDO: Indoleamine 2,3-dioxygenase
IG: Interstitial glucose
LC-MS: Liquid chromatography–mass spectrometry
LFP: Lyophilized feces preparation
LPS: Lipopolysaccharide
MS: Mass spectrometry
MS-DIAL: Mass spectrometry–data independent analysis software
MSEA: Metabolite set enrichment analysis
MSI: Metabolomics standards initiative
NES: Normalized enrichment score
NIST: National institute of standards and technology
PCA: Principal component analysis
Q–Q: Quantile–quantile (plot)
R: R (statistical computing environment)
SAS: Statistical analysis system
SCFA: Short-chain fatty acid
SCFAs: Short-chain fatty acids
SCD1: Stearoyl-CoA desaturase-1
SCD1_16: Stearoyl-CoA desaturase index based on 16:1/16:0
SCD1_18: Stearoyl-CoA desaturase index based on 18:1/18:0
SD: Standard deviation
TCA: Tricarboxylic acid (cycle)
UHPLC: Ultra-high-performance liquid chromatography
