# Supplementary figures and images for: Serum metabolomics of diabetic dogs treated with daily administration of a commercially available lyophilized feces preparation
Source: Vet Res Commun. 2026 Mar 27;50(3):229. doi: 10.1007/s11259-026-11181-9 (PMC13031211; doi:10.1007/s11259-026-11181-9)

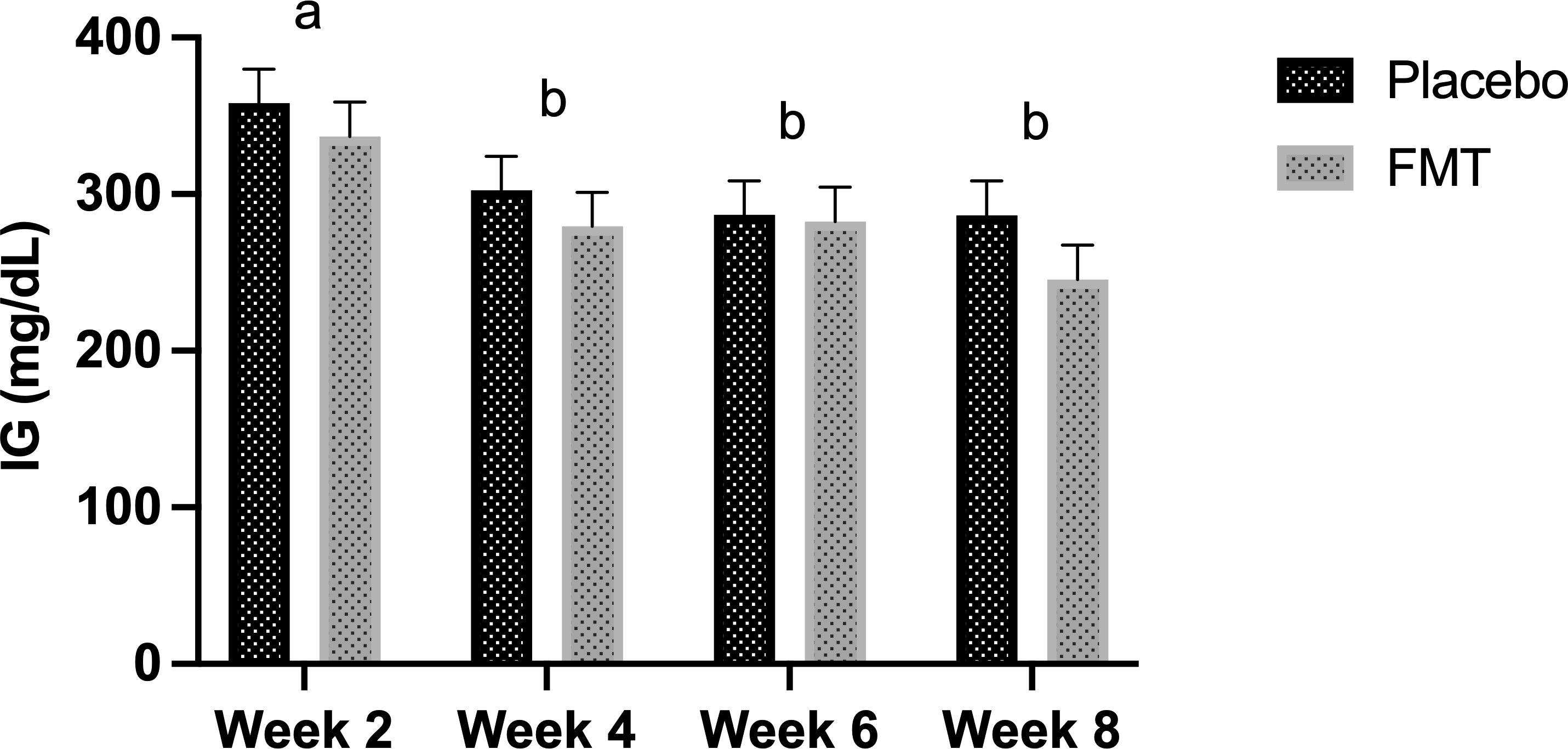

Supplement: Supplementary file 1 — Supplementary Material 1. [file 11259_2026_11181_MOESM1_ESM.tif]

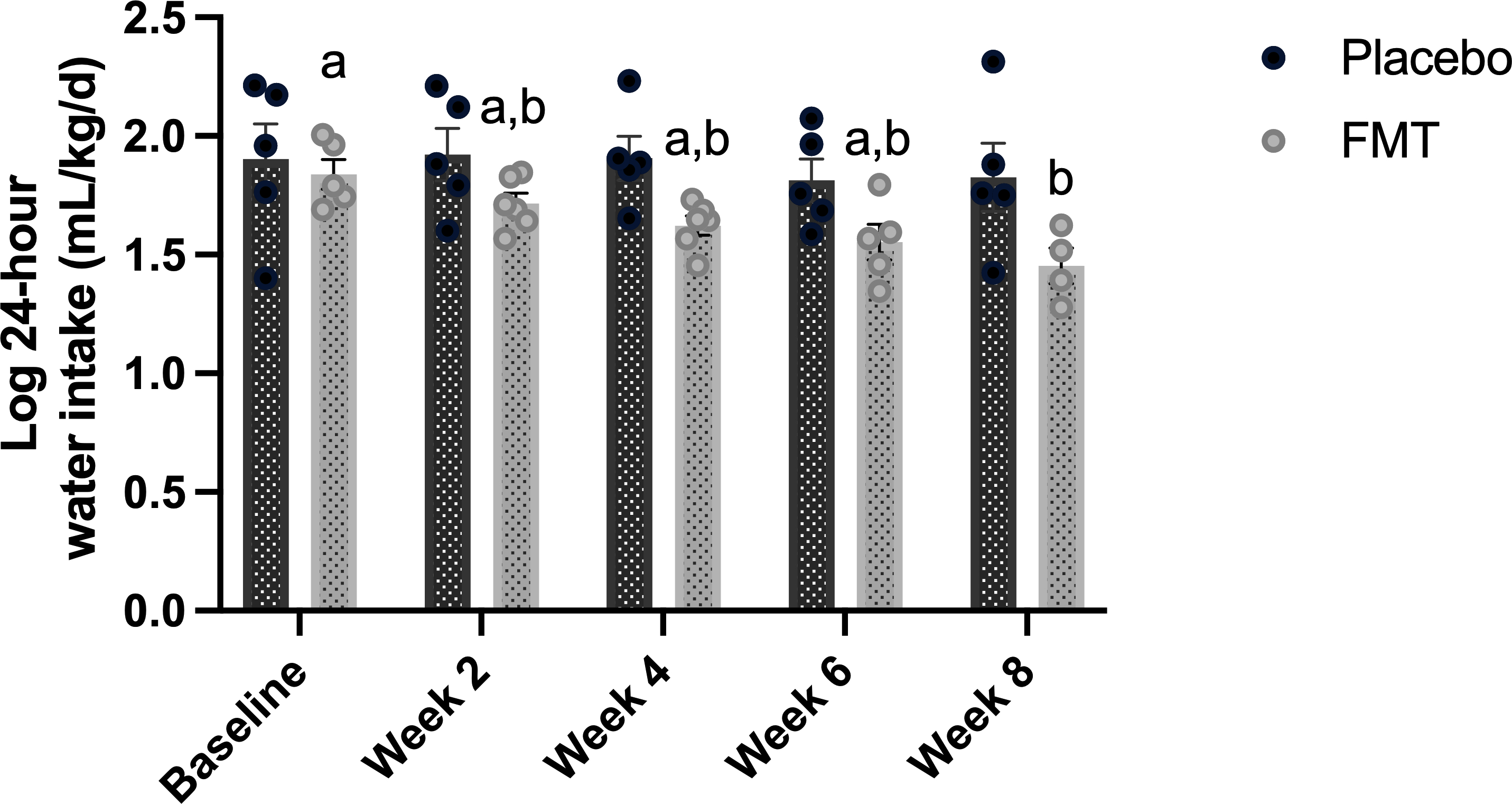

Supplement: Supplementary file 2 — Supplementary Material 2. [file 11259_2026_11181_MOESM2_ESM.tif]
