# Supplementary material for: Serum metabolomics of diabetic dogs treated with daily administration of a commercially available lyophilized feces preparation
Source: Vet Res Commun. 2026 Mar 27;50(3):229. doi: 10.1007/s11259-026-11181-9 (PMC13031211; doi:10.1007/s11259-026-11181-9)
Supplement: Supplementary file 7 — Supplementary Material 7. [file 11259_2026_11181_MOESM7_ESM.docx]

**Supplementary File 2**

**Table 1.** GCMS Metabolite Contrast Output.

| Biochemical | SuperPathway | SubPathway | contrast | estimate | SE | q.value | Log2FC |
| --- | --- | --- | --- | --- | --- | --- | --- |
| 9_12_Octadecadienoic_acid | Lipids and lipid-like molecules | Fatty Acyls | placebo - FMT | -0.5399725 | 0.17745853 | 0.01240297 | -0.7790156 |
| 11_Octadecenoic_acid | Lipids and lipid-like molecules | Fatty Acyls | placebo - FMT | -0.6632393 | 0.2258409 | 0.01486787 | -0.956852 |
| sucrose | Organic oxygen compounds | Organooxygen compounds | placebo - FMT | 1.10872293 | 0.44314347 | 0.03134194 | 1.59954907 |
| Citric_acid | Organic acids and derivatives | Carboxylic acids and derivatives | placebo - FMT | 0.33335369 | 0.14152151 | 0.04026127 | 0.48092771 |
| methyl_alanine | Organoheterocyclic compounds | Benzodiazepines | placebo - FMT | -0.8748794 | 0.4090026 | 0.0581274 | -1.2621842 |
| palmitoleic_acid | Lipids and lipid-like molecules | Fatty Acyls | placebo - FMT | -1.7219679 | 0.82739378 | 0.06406918 | -2.4842745 |
| 1_Monooctadecanoylglycerol | Lipids and lipid-like molecules | Glycerolipids | placebo - FMT | -0.3348336 | 0.1623945 | 0.06618141 | -0.4830627 |
| uracil | Organoheterocyclic compounds | Diazines | placebo - FMT | 0.52038736 | 0.27948854 | 0.09222275 | 0.75076026 |
| Oxalic_acid | Organic acids and derivatives | Carboxylic acids and derivatives | placebo - FMT | 0.59796842 | 0.34808488 | 0.11656941 | 0.86268608 |
| Glycerol | Organic oxygen compounds | Organooxygen compounds | placebo - FMT | -0.3602989 | 0.22673498 | 0.14312744 | -0.5198014 |
| Inositol__myo | Organic oxygen compounds | Organooxygen compounds | placebo - FMT | 0.22092374 | 0.1403157 | 0.14645429 | 0.31872558 |
| THREONIC_ACID | Organic oxygen compounds | Organooxygen compounds | placebo - FMT | 0.42625757 | 0.27111448 | 0.14696943 | 0.61495968 |
| HYDROQUINONE | Benzenoids | Phenols | placebo - FMT | 1.70597341 | 1.13355586 | 0.16324345 | 2.46119938 |
| isoleucine | Organic acids and derivatives | Carboxylic acids and derivatives | placebo - FMT | -0.4074276 | 0.27733509 | 0.17255404 | -0.5877938 |
| N_acetyltryptophan | Organic acids and derivatives | Carboxylic acids and derivatives | placebo - FMT | 0.3275415 | 0.22615394 | 0.17814792 | 0.47254249 |
| hexadecanoic_acid__palmitic_ | Lipids and lipid-like molecules | Fatty Acyls | placebo - FMT | -0.2203612 | 0.16047944 | 0.19971107 | -0.3179141 |
| octadecanoic_acid__stearic_ | Lipids and lipid-like molecules | Fatty Acyls | placebo - FMT | -0.152709 | 0.11224162 | 0.20353511 | -0.2203125 |
| Hypoxanthine | Organoheterocyclic compounds | Imidazopyrimidines | placebo - FMT | 0.76058415 | 0.5804203 | 0.2193579 | 1.09729098 |
| oleic_acid | Lipids and lipid-like molecules | Fatty Acyls | placebo - FMT | -0.4612741 | 0.35474569 | 0.22266848 | -0.6654779 |
| Benzoic_acid | Benzenoids | Benzene and substituted derivatives | placebo - FMT | -0.5223576 | 0.41910994 | 0.24104164 | -0.7536028 |
| Glycolic_acid | Organic acids and derivatives | Hydroxy acids and derivatives | placebo - FMT | -0.1366862 | 0.11349205 | 0.25617851 | -0.1971965 |
| Cholesterol | Lipids and lipid-like molecules | Steroids and steroid derivatives | placebo - FMT | -0.1043344 | 0.08718782 | 0.25903803 | -0.1505227 |
| Butanoic_acid__2_hydroxy | Organic acids and derivatives | Hydroxy acids and derivatives | placebo - FMT | -0.3423926 | 0.29512865 | 0.27293743 | -0.4939681 |
| PYRUVIC_ACID | Organic acids and derivatives | Keto acids and derivatives | placebo - FMT | 0.71244519 | 0.62383549 | 0.28004517 | 1.02784114 |
| Glutamic_acid | Organic acids and derivatives | Carboxylic acids and derivatives | placebo - FMT | 0.34698543 | 0.30740801 | 0.28535582 | 0.50059416 |
| Erythritol | Organic oxygen compounds | Organooxygen compounds | placebo - FMT | 0.38461155 | 0.35348307 | 0.30209554 | 0.55487718 |
| Decanoic_acid | Lipids and lipid-like molecules | Fatty Acyls | placebo - FMT | 0.37288927 | 0.36678732 | 0.33329211 | 0.53796551 |
| Threonine | Organic acids and derivatives | Carboxylic acids and derivatives | placebo - FMT | -0.2147384 | 0.21150972 | 0.33391392 | -0.309802 |
| Erythronic_acid | Organic oxygen compounds | Organooxygen compounds | placebo - FMT | 0.24790432 | 0.25820789 | 0.35964555 | 0.35765033 |
| valine | Organic acids and derivatives | Carboxylic acids and derivatives | placebo - FMT | -0.2181956 | 0.22738862 | 0.35989684 | -0.3147898 |
| ribose | Organic oxygen compounds | Organooxygen compounds | placebo - FMT | 0.24115286 | 0.25777708 | 0.37156671 | 0.34791003 |
| glycine | Organic acids and derivatives | Carboxylic acids and derivatives | placebo - FMT | -0.1484004 | 0.16343882 | 0.38524395 | -0.2140965 |
| Malic_acid | Organic acids and derivatives | Hydroxy acids and derivatives | placebo - FMT | 0.50139659 | 0.57493953 | 0.40361429 | 0.72336238 |
| UREA | Organic acids and derivatives | Organic carbonic acids and derivatives | placebo - FMT | 0.1429001 | 0.17544858 | 0.43433509 | 0.20616126 |
| Succinic_acid | Organic acids and derivatives | Carboxylic acids and derivatives | placebo - FMT | -0.0900014 | 0.11489472 | 0.45158184 | -0.1298446 |
| 5_8_11_14_17_Eicosapentaenoic_acid | Lipids and lipid-like molecules | Fatty Acyls | placebo - FMT | -0.0836505 | 0.11600408 | 0.48736438 | -0.1206821 |
| fructose | Organic oxygen compounds | Organooxygen compounds | placebo - FMT | -0.263782 | 0.36609203 | 0.48769701 | -0.3805569 |
| Serine | Organic acids and derivatives | Carboxylic acids and derivatives | placebo - FMT | -0.1721701 | 0.24269407 | 0.49427692 | -0.2483889 |
| Inosine | Nucleosides, nucleotides, and analogues | Purine nucleosides | placebo - FMT | -0.1137581 | 0.16183145 | 0.49812974 | -0.1641182 |
| 1_5_anhydro_D_sorbitol | Organic oxygen compounds | Organooxygen compounds | placebo - FMT | 0.49087563 | 0.73653229 | 0.52019078 | 0.70818384 |
| Butanoic_acid__2_4_dihydroxy | Organic acids and derivatives | Hydroxy acids and derivatives | placebo - FMT | -0.3460522 | 0.52061806 | 0.52127829 | -0.4992478 |
| N_Acetylglutamic_acid | Organic acids and derivatives | Carboxylic acids and derivatives | placebo - FMT | 0.19525247 | 0.31228012 | 0.54581118 | 0.28168976 |
| 1_Monohexadecanoylglycerol | Lipids and lipid-like molecules | Glycerolipids | placebo - FMT | -0.0719621 | 0.11997058 | 0.56196341 | -0.1038193 |
| Azelaic_acid | Lipids and lipid-like molecules | Fatty Acyls | placebo - FMT | 0.4097927 | 0.7346743 | 0.58925697 | 0.5912059 |
| methionine | Organic acids and derivatives | Carboxylic acids and derivatives | placebo - FMT | -0.4119418 | 0.74085677 | 0.59041088 | -0.5943063 |
| lactic_acid | Organic acids and derivatives | Hydroxy acids and derivatives | placebo - FMT | 0.10109326 | 0.20246276 | 0.62835718 | 0.14584674 |
| Glutamine | Organic acids and derivatives | Carboxylic acids and derivatives | placebo - FMT | -0.0419336 | 0.08555987 | 0.63463168 | -0.0604974 |
| leucine | Organic acids and derivatives | Carboxylic acids and derivatives | placebo - FMT | -0.1389253 | 0.31278209 | 0.6663841 | -0.2004269 |
| 4_Hydroxyproline | Organic acids and derivatives | Carboxylic acids and derivatives | placebo - FMT | 0.2569329 | 0.59019906 | 0.67256693 | 0.37067582 |
| CYSTEINE | Organic acids and derivatives | Carboxylic acids and derivatives | placebo - FMT | -0.1528505 | 0.36043553 | 0.68049139 | -0.2205167 |
| lactamide | Organic oxygen compounds | Organooxygen compounds | placebo - FMT | 0.12172844 | 0.28950908 | 0.68303818 | 0.17561702 |
| pyrophosphate__4_1_ | Mixed metal/non-metal compounds | Alkali metal oxoanionic compounds | placebo - FMT | 0.22646673 | 0.53865851 | 0.68306485 | 0.32672243 |
| glyoxylic_acid | Organic acids and derivatives | Carboxylic acids and derivatives | placebo - FMT | -0.1001077 | 0.23845234 | 0.68349235 | -0.1444248 |
| arabinose | Organic oxygen compounds | Organooxygen compounds | placebo - FMT | 0.31457961 | 0.75220821 | 0.68463395 | 0.45384244 |
| Dodecanoic_ACID | Lipids and lipid-like molecules | Fatty Acyls | placebo - FMT | 0.02498517 | 0.06276945 | 0.69896105 | 0.03604598 |
| Phenylalanine | Organic acids and derivatives | Carboxylic acids and derivatives | placebo - FMT | -0.0471759 | 0.11974692 | 0.7018783 | -0.0680604 |
| Alanine | Organic acids and derivatives | Carboxylic acids and derivatives | placebo - FMT | -0.0457278 | 0.11665765 | 0.70329482 | -0.0659713 |
| Monomethylphosphate | Organic acids and derivatives | Organic phosphoric acids and derivatives | placebo - FMT | 0.23298871 | 0.65335399 | 0.72879779 | 0.33613166 |
| Glutaric_acid__2_hydroxy | Organic acids and derivatives | Hydroxy acids and derivatives | placebo - FMT | 0.34913564 | 1.05228733 | 0.74689881 | 0.50369626 |
| ethanolamine | Organic nitrogen compounds | Organonitrogen compounds | placebo - FMT | -0.0782582 | 0.25135702 | 0.76193351 | -0.1129028 |
| mannose | Organic oxygen compounds | Organooxygen compounds | placebo - FMT | -0.1183537 | 0.38111481 | 0.76252182 | -0.1707482 |
| Fumaric_acid | Organic acids and derivatives | Carboxylic acids and derivatives | placebo - FMT | 0.05395737 | 0.19589076 | 0.78858124 | 0.07784402 |
| arabitol | Organic oxygen compounds | Organooxygen compounds | placebo - FMT | 0.04617147 | 0.20804262 | 0.82883294 | 0.06661135 |
| Glucose | Organic oxygen compounds | Organooxygen compounds | placebo - FMT | -0.0582681 | 0.27705215 | 0.8376455 | -0.0840631 |
| valeramide | Lipids and lipid-like molecules | Fatty Acyls | placebo - FMT | -0.0352914 | 0.1698661 | 0.83958639 | -0.0509147 |
| 2_Keto_L_gluconic_acid | Organic oxygen compounds | Organooxygen compounds | placebo - FMT | -0.117472 | 0.60393134 | 0.84966968 | -0.1694762 |
| Glycerol_3_p | Lipids and lipid-like molecules | Glycerophospholipids | placebo - FMT | -0.0419815 | 0.22543455 | 0.85599192 | -0.0605665 |
| proline | Organic acids and derivatives | Carboxylic acids and derivatives | placebo - FMT | -0.1561861 | 0.88663888 | 0.8636882 | -0.2253289 |
| threose | Organic oxygen compounds | Organooxygen compounds | placebo - FMT | -0.0908928 | 0.53171147 | 0.86767726 | -0.1311306 |
| Butanoic_acid__3_hydroxy | Organic acids and derivatives | Hydroxy acids and derivatives | placebo - FMT | 0.05865691 | 0.38465582 | 0.88183186 | 0.08462403 |
| Heptadecanoic_acid | Lipids and lipid-like molecules | Fatty Acyls | placebo - FMT | -0.0617195 | 0.42755571 | 0.88808843 | -0.0890425 |
| Butanoic_acid__2_methyl_2_hydroxy | Lipids and lipid-like molecules | Fatty Acyls | placebo - FMT | 0.05994921 | 0.86916544 | 0.9463706 | 0.08648842 |
| maltose | Organic oxygen compounds | Organooxygen compounds | placebo - FMT | 0.01188126 | 0.46884863 | 0.98028126 | 0.01714103 |

Log2FC values are provided for descriptive context; statistical inference is based on longitudinal mixed-effects model estimates.

**Table 2.** GCMS Metabolite FgseaRes

| \| pathway \| SuperPathway \| pval \| padj \| log2err \| ES \| NES \| leadingEdge \| \| --- \| --- \| --- \| --- \| --- \| --- \| --- \| --- \| \| Fatty Acyls \| Lipids and lipid-like molecules \| 0.011804522 \| 0.059022609 \| 0.380730401 \| -0.84012409 \| -1.538046706 \| c(“palmitoleic_acid”, “11_Octadecenoic_acid”, “9_12_Octadecadienoic_acid”, “oleic_acid”, “exadecenoic_acid__palmitic_”, “octadecanoic_acid__stearic_”) \| \| Glycerolipids \| Lipids and lipid-like molecules \| 0.186192469 \| 0.310320781 \| 0.146416238 \| -0.900036649 \| -1.245880315 \| 1_Monooctadecanoylglycerol \| \| Organooxygen compounds \| Organic oxygen compounds \| 0.134969325 \| 0.310320781 \| 0.172324345 \| 0.669455098 \| 1.317130238 \| c(“sucrose”, “THREONIC_ACID”, “Erythritol”, “Inositol__myo”, “1_5_anhydro_D_sorbitol”, “Erythronic_acid”, “ribose”, “arabinose”) \| \| Carboxylic acids and derivatives \| Organic acids and derivatives \| 0.552795031 \| 0.690993789 \| 0.075694631 \| 0.451628293 \| 0.931822244 \| c(“Oxalic_acid”, “Citric_acid”, “N_acetyltryptophan”, “Glutamic_acid”) \| \| Hydroxy acids and derivatives \| Organic acids and derivatives \| 0.932914046 \| 0.932914046 \| 0.051011413 \| -0.356226332 \| -0.577107082 \| c(“Butanoic_acid__2_hydroxy”, “Butanoic_acid__2_4_dihydroxy”, “Glycolic_acid”) \| |
| --- | --- | --- | --- | --- | --- | --- | --- | --- | --- | --- | --- | --- | --- | --- | --- | --- | --- | --- | --- | --- | --- | --- | --- | --- | --- | --- | --- | --- | --- | --- | --- | --- | --- | --- | --- | --- | --- | --- | --- | --- | --- | --- | --- | --- | --- | --- | --- | --- |

**Table 3.** LCMS Metabolite Contrast Output

| Biochemical | SuperPathway | SubPathway | contrast | estimate | SE | q.value | | Log2FC |
| --- | --- | --- | --- | --- | --- | --- | --- | --- |
| DAIDZEIN_4__SULFATE | Phenylpropanoids and polyketides | Isoflavonoids | placebo - FMT | 2.51204634 | 0.58656726 | 0.00160475 | 3.6241168 | |
| LINOLEIC_ACID | Lipids and lipid-like molecules | Fatty Acyls | placebo - FMT | -0.5604702 | 0.18562902 | 0.01291082 | -0.8085876 | |
| RAC__1R_3AR_6AS__OCTAHYDROCYCLOPENTA_C_PYRROLE_1_CARBOXYLIC_ACID | Organic oxygen compounds | Organooxygen compounds | placebo - FMT | 0.46891457 | 0.16263338 | 0.01629421 | 0.67650073 | |
| 1_ACETAMIDOCYCLOPENTANE_1_CARBOXYLIC_ACID | Organic acids and derivatives | Carboxylic acids and derivatives | placebo - FMT | -0.4565138 | 0.16268684 | 0.01859925 | -0.6586102 | |
| 2_PIPERIDINECARBOXAMIDE | Organic acids and derivatives | Carboxylic acids and derivatives | placebo - FMT | -0.4814793 | 0.17333296 | 0.01952519 | -0.6946278 | |
| LYSINE | Organic acids and derivatives | Carboxylic acids and derivatives | placebo - FMT | -0.4564057 | 0.1661051 | 0.0205595 | -0.6584543 | |
| METHACHOLINE_CATION | Organic nitrogen compounds | Organonitrogen compounds | placebo - FMT | 1.02462021 | 0.37493936 | 0.02109316 | 1.4782145 | |
| HOMOARGININE | Organic acids and derivatives | Carboxylic acids and derivatives | placebo - FMT | -0.920861 | 0.33948728 | 0.0218395 | -1.3285216 | |
| GLYCERATE | Organic oxygen compounds | Organooxygen compounds | placebo - FMT | 0.4181662 | 0.15590477 | 0.02300607 | 0.6032863 | |
| D_____ISOASCORBIC_ACID | Organoheterocyclic compounds | Dihydrofurans | placebo - FMT | 0.627862 | 0.24066427 | 0.0260913 | 0.9058134 | |
| STEAROYL_L_CARNITINE | Lipids and lipid-like molecules | Fatty Acyls | placebo - FMT | 0.69043283 | 0.26562294 | 0.02652348 | 0.99608402 | |
| CHOLINE_CHLORIDE | Organic nitrogen compounds | Organonitrogen compounds | placebo - FMT | 0.4308334 | 0.16586626 | 0.02660654 | 0.62156122 | |
| 2_AMINOACETOPHENONE | Organic oxygen compounds | Organooxygen compounds | placebo - FMT | 0.37124566 | 0.14409138 | 0.02758331 | 0.53559428 | |
| ALLANTOIN | Organoheterocyclic compounds | Azoles | placebo - FMT | 0.26595772 | 0.11670562 | 0.04587324 | 0.38369588 | |
| XANTHINE | Organoheterocyclic compounds | Imidazopyrimidines | placebo - FMT | 0.49589073 | 0.2265991 | 0.05347997 | 0.71541909 | |
| 3_ETHYLPHENOL | Benzenoids | Phenols | placebo - FMT | 1.87966541 | 0.86407487 | 0.05467348 | 2.71178396 | |
| LAETISARIC_ACID | Lipids and lipid-like molecules | Fatty Acyls | placebo - FMT | -0.6270206 | 0.2910425 | 0.05664283 | -0.9045996 | |
| 3_4_5_TRIMETHOXYPHENOL | Benzenoids | Phenols | placebo - FMT | 1.2372162 | 0.57716754 | 0.0576838 | 1.78492568 | |
| PSEUDOURIDINE | Nucleosides, nucleotides, and analogues | Nucleoside and nucleotide analogues | placebo - FMT | 0.2542089 | 0.11934314 | 0.05901459 | 0.36674592 | |
| N_ACETYLNEURAMINATE | Organic oxygen compounds | Organooxygen compounds | placebo - FMT | 0.60773409 | 0.30516135 | 0.07443235 | 0.87677496 | |
| N_ACETYL_D_TRYPTOPHAN | Organic acids and derivatives | Carboxylic acids and derivatives | placebo - FMT | 0.62111466 | 0.31502498 | 0.07693499 | 0.89607904 | |
| SUCROSE | Organic oxygen compounds | Organooxygen compounds | placebo - FMT | 0.66371083 | 0.34233968 | 0.08124907 | 0.95753232 | |
| N__4_BROMOPHENYL_ACRYLAMIDE | Benzenoids | Benzene and substituted derivatives | placebo - FMT | 0.57845601 | 0.30055276 | 0.08316845 | 0.83453562 | |
| 4__2_FURYL__2_4_DIOXOBUTANOIC_ACID | Organic acids and derivatives | Keto acids and derivatives | placebo - FMT | 0.47243721 | 0.24605035 | 0.08379738 | 0.68158283 | |
| 5__ACETYLAMINO__2_HYDROXYBENZOIC_ACID | Benzenoids | Benzene and substituted derivatives | placebo - FMT | 1.44922858 | 0.75936522 | 0.08541983 | 2.09079489 | |
| C12_AS__TENTATIVE_ | Organic acids and derivatives | Organic sulfuric acids and derivatives | placebo - FMT | 0.24990931 | 0.13222113 | 0.08804949 | 0.36054293 | |
| 3_Hydroxy_3__methoxycarbonyl_pentanedioic_acid | Organic acids and derivatives | Carboxylic acids and derivatives | placebo - FMT | 0.45001286 | 0.23900954 | 0.08910867 | 0.64923132 | |
| L_KYNURENINE | Organic oxygen compounds | Organooxygen compounds | placebo - FMT | 0.52312368 | 0.2857657 | 0.09707993 | 0.75470794 | |
| 5_METHOXYSALICYLIC_ACID_SULFATE | Undefined | Undefined | placebo - FMT | 0.9127533 | 0.50079479 | 0.09835678 | 1.31682465 | |
| TRP_ASP | Organic acids and derivatives | Carboxylic acids and derivatives | placebo - FMT | 0.89745568 | 0.49872331 | 0.10213436 | 1.29475486 | |
| N__4_CHLOROBENZOYL_TRYPTOPHAN | Benzenoids | Benzene and substituted derivatives | placebo - FMT | 0.35614244 | 0.20029653 | 0.10575703 | 0.51380493 | |
| 4_METHOXYNAPHTHALEN_1_AMINE | Benzenoids | Naphthalenes | placebo - FMT | 0.34089734 | 0.19554434 | 0.11188193 | 0.4918109 | |
| L_TRYPTOPHAN | Organoheterocyclic compounds | Indoles and derivatives | placebo - FMT | 0.38453089 | 0.2206122 | 0.11193759 | 0.55476081 | |
| 5_OXO_1_PROPYL_2_PYRROLIDINEACETIC_ACID | Organoheterocyclic compounds | Pyrrolidines | placebo - FMT | 0.28742427 | 0.16527914 | 0.11266221 | 0.41466556 | |
| 9__2_3_DIHYDROXYPROPOXY__9_OXONONANOIC_ACID | Lipids and lipid-like molecules | Fatty Acyls | placebo - FMT | 0.38895667 | 0.22972581 | 0.12129842 | 0.56114586 | |
| SPERMIDINE | Organic nitrogen compounds | Organonitrogen compounds | placebo - FMT | 0.48593246 | 0.28715465 | 0.12147398 | 0.70105235 | |
| 1_ACETYLPIPERIDINE_2_CARBOXYLIC_ACID | Organic acids and derivatives | Carboxylic acids and derivatives | placebo - FMT | 0.47011478 | 0.27855762 | 0.12236472 | 0.67823227 | |
| HOMOCITRULLINE | Organic acids and derivatives | Carboxylic acids and derivatives | placebo - FMT | 0.4145166 | 0.24818003 | 0.12582989 | 0.59802104 | |
| 1_2_DIHYDROSPIRO_INDOLE_3_3__PYRROLIDINE__2_ONE | Organic acids and derivatives | Carboxylic acids and derivatives | placebo - FMT | 0.31398512 | 0.18909598 | 0.12780867 | 0.45298478 | |
| PHE_TRP | Organic acids and derivatives | Carboxylic acids and derivatives | placebo - FMT | 0.353571 | 0.21534583 | 0.13164792 | 0.51009514 | |
| GLUTAMATE | Organic acids and derivatives | Carboxylic acids and derivatives | placebo - FMT | 0.35829292 | 0.22453166 | 0.14163219 | 0.51690742 | |
| ELAIDATE | Lipids and lipid-like molecules | Fatty Acyls | placebo - FMT | -0.5077864 | 0.31891642 | 0.14241862 | -0.732581 | |
| 2_2_DIMETHYL_4_PENTENOIC_ACID | Lipids and lipid-like molecules | Fatty Acyls | placebo - FMT | 0.53536437 | 0.33664121 | 0.14284879 | 0.77236753 | |
| PALMITOYL_L_CARNITINE | Lipids and lipid-like molecules | Fatty Acyls | placebo - FMT | 0.38562124 | 0.24287998 | 0.14343727 | 0.55633386 | |
| CITRIC_ACID | Organic acids and derivatives | Carboxylic acids and derivatives | placebo - FMT | 0.58068136 | 0.36594917 | 0.14364564 | 0.83774613 | |
| 5_METHYL_2__DEOXYCYTIDINE | Benzenoids | Triphenyl compounds | placebo - FMT | -0.5090462 | 0.32158367 | 0.1445183 | -0.7343985 | |
| DL_ISOCITRIC_ACID_LACTONE | Organic acids and derivatives | Carboxylic acids and derivatives | placebo - FMT | 0.58087572 | 0.36746732 | 0.14501522 | 0.83802652 | |
| N_ACETYL_L_GLUTAMIC_ACID | Organic acids and derivatives | Carboxylic acids and derivatives | placebo - FMT | 0.29872563 | 0.19066894 | 0.1482485 | 0.43096999 | |
| GLUCONATE | Organic oxygen compounds | Organooxygen compounds | placebo - FMT | 0.35324008 | 0.22577368 | 0.14874911 | 0.50961771 | |
| ANILINE | Benzenoids | Benzene and substituted derivatives | placebo - FMT | 0.3025328 | 0.1945035 | 0.15090177 | 0.43646257 | |
| 12S_HYDROXY_5Z_8E_10E_HEPTADECATRIENOIC_ACID | Lipids and lipid-like molecules | Fatty Acyls | placebo - FMT | 0.43152203 | 0.27829502 | 0.15204445 | 0.6225547 | |
| 2_NAPHTHYLAMINE | Benzenoids | Naphthalenes | placebo - FMT | 0.34044368 | 0.21961238 | 0.15213714 | 0.4911564 | |
| 3_HYDROXY_3___4_7_7_TRIMETHYL_3_BICYCLO_221_HEPTANYL_OXYCARBONYL_PENTANEDIOIC_ACID | Undefined | Undefined | placebo - FMT | 0.55251083 | 0.35744829 | 0.1532103 | 0.79710463 | |
| 3_FORMYLINDOLE | Organoheterocyclic compounds | Indoles and derivatives | placebo - FMT | 0.33472917 | 0.21928682 | 0.15788595 | 0.48291212 | |
| PYROGLU_PRO | Organic acids and derivatives | Carboxylic acids and derivatives | placebo - FMT | 0.43213713 | 0.28402877 | 0.15911705 | 0.62344209 | |
| INDOLE | Organoheterocyclic compounds | Indoles and derivatives | placebo - FMT | 0.33886317 | 0.22277687 | 0.15920811 | 0.48887621 | |
| 3__5__2_METHYLPROPYL__3_6_DIOXOPIPERAZIN_2_YL_PROPANOIC_ACID | Organic acids and derivatives | Carboximidic acids and derivatives | placebo - FMT | 0.55588367 | 0.36627624 | 0.16005863 | 0.80197061 | |
| ETHYL_4_HYDROXYQUINOLINE_2_CARBOXYLATE | Organoheterocyclic compounds | Quinolines and derivatives | placebo - FMT | -0.6809836 | 0.44985976 | 0.16103023 | -0.9824517 | |
| N_OCTYL_SULFATE | Organic acids and derivatives | Organic sulfuric acids and derivatives | placebo - FMT | 0.41160548 | 0.27219034 | 0.16142382 | 0.59382118 | |
| PROPYLPARABEN | Benzenoids | Benzene and substituted derivatives | placebo - FMT | 0.36408904 | 0.24263944 | 0.16437094 | 0.52526945 | |
| 3_ISOBUTYLPENTANEDIOIC_ACID | Lipids and lipid-like molecules | Fatty Acyls | placebo - FMT | 0.25062647 | 0.16761044 | 0.16571127 | 0.36157757 | |
| PIPECOLATE | Organic acids and derivatives | Carboxylic acids and derivatives | placebo - FMT | -0.3418017 | 0.22964928 | 0.16749655 | -0.4931156 | |
| _3R__5__2_FLUOROPHENYL__3__1H_INDOL_3_YLMETHYL__1_METHYL_3H_1_4_BENZODIAZEPIN_2_ONE | Organoheterocyclic compounds | Benzodiazepines | placebo - FMT | 0.34301703 | 0.23431835 | 0.17393687 | 0.49486897 | |
| N_EPSILON_ACETYLLYSINE | Organic acids and derivatives | Carboxylic acids and derivatives | placebo - FMT | 0.19085864 | 0.13082253 | 0.17527352 | 0.27535082 | |
| ETHYL_SULFATE | Organic acids and derivatives | Organic sulfuric acids and derivatives | placebo - FMT | 0.7930102 | 0.5459638 | 0.17700865 | 1.14407188 | |
| ISOBUTYRYL_L_CARNITINE | Lipids and lipid-like molecules | Fatty Acyls | placebo - FMT | 0.45791507 | 0.31724437 | 0.17948961 | 0.6606318 | |
| GEMFIBROZIL | Benzenoids | Phenol ethers | placebo - FMT | 0.24875347 | 0.17433567 | 0.1840874 | 0.3588754 | |
| PARAXANTHINE | Organoheterocyclic compounds | Imidazopyrimidines | placebo - FMT | 0.62917878 | 0.4418777 | 0.18492866 | 0.90771311 | |
| THROMBOXANE_B2 | Lipids and lipid-like molecules | Fatty Acyls | placebo - FMT | 0.41834686 | 0.29421299 | 0.18548112 | 0.60354694 | |
| GABAPENTIN | Organic acids and derivatives | Carboxylic acids and derivatives | placebo - FMT | -1.0894371 | 0.7746265 | 0.18991107 | -1.5717255 | |
| 11_15_DIOXO_9S_HYDROXY_5Z_PROSTENOIC_ACID | Lipids and lipid-like molecules | Fatty Acyls | placebo - FMT | 0.45227003 | 0.32430929 | 0.19335165 | 0.65248772 | |
| TRAMADOL_N_OXIDE | Benzenoids | Phenol ethers | placebo - FMT | 1.5205896 | 1.09061107 | 0.19344222 | 2.19374707 | |
| 5_KETO_D_GLUCONIC_ACID | Organic acids and derivatives | Hydroxy acids and derivatives | placebo - FMT | 0.32596418 | 0.23479548 | 0.19519629 | 0.47026691 | |
| ISOVALERYL_L_CARNITINE | Lipids and lipid-like molecules | Fatty Acyls | placebo - FMT | 0.39554591 | 0.28665491 | 0.19769585 | 0.57065212 | |
| ALPHA_N_ACETYL_GLUTAMINE | Organic acids and derivatives | Carboxylic acids and derivatives | placebo - FMT | 0.30406802 | 0.22192235 | 0.20061206 | 0.43867743 | |
| DIETHYL_2___2_HYDROXYANILINO_METHYLENE_MALONATE | Undefined | Undefined | placebo - FMT | 0.20703115 | 0.15110426 | 0.20062227 | 0.29868282 | |
| ADENINE | Organoheterocyclic compounds | Imidazopyrimidines | placebo - FMT | -0.5955269 | 0.43600436 | 0.20190891 | -0.8591638 | |
| 3_HYDROXY_3_METHYLGLUTARATE | Lipids and lipid-like molecules | Fatty Acyls | placebo - FMT | 0.72812668 | 0.53340756 | 0.20216002 | 1.05046475 | |
| 3_FURANCARBOXYLIC_ACID__TETRAHYDRO_4_METHYLENE_2_OCTYL_5_OXO____2R_3S__REL_ | Organoheterocyclic compounds | Lactones | placebo - FMT | 0.24697497 | 0.18134866 | 0.20312532 | 0.35630957 | |
| 5_AMINOPENTANAMIDE | Organic acids and derivatives | Carboxylic acids and derivatives | placebo - FMT | -0.7268864 | 0.53766009 | 0.20617738 | -1.0486754 | |
| N__3_PIPERIDINYL_ACETAMIDE | Organoheterocyclic compounds | Piperidines | placebo - FMT | 0.50928232 | 0.37895941 | 0.20867767 | 0.73473908 | |
| CAFFEINE | Organoheterocyclic compounds | Imidazopyrimidines | placebo - FMT | 0.21146144 | 0.1573933 | 0.20879458 | 0.30507437 | |
| 1_PENTADECANOYL_SN_GLYCERO_3_PHOSPHOCHOLINE | Lipids and lipid-like molecules | Glycerophospholipids | placebo - FMT | 0.25219609 | 0.18901673 | 0.21170738 | 0.36384205 | |
| RAC_N_N_DIDESMETHYL_O_DESMETHYLVENLAFAXINE_GLUCURONIDE | Organic oxygen compounds | Organooxygen compounds | placebo - FMT | 1.92851725 | 1.44722371 | 0.21224188 | 2.78226227 | |
| 2_DEC_9_ENYLPENTANEDIOIC_ACID | Lipids and lipid-like molecules | Fatty Acyls | placebo - FMT | -0.2224989 | 0.1677948 | 0.21432344 | -0.320998 | |
| THREONIC_ACID | Organic oxygen compounds | Organooxygen compounds | placebo - FMT | 0.20892968 | 0.15827891 | 0.21624899 | 0.30142181 | |
| TETRADECANEDIOIC_ACID | Lipids and lipid-like molecules | Fatty Acyls | placebo - FMT | -0.5666355 | 0.42964238 | 0.21662103 | -0.8174823 | |
| LEVETIRACETAM | Organic acids and derivatives | Carboxylic acids and derivatives | placebo - FMT | 0.27116968 | 0.20564964 | 0.21670274 | 0.39121516 | |
| 1__1Z_HEXADECENYL__SN_GLYCERO_3_PHOSPHOCHOLINE | Lipids and lipid-like molecules | Glycerophospholipids | placebo - FMT | -0.2876112 | 0.21981031 | 0.21999368 | -0.4149352 | |
| S_PHENYLMERCAPTURIC_ACID | Organic acids and derivatives | Carboxylic acids and derivatives | placebo - FMT | 0.81430669 | 0.62591594 | 0.22244156 | 1.17479622 | |
| HEXADECANEDIOIC_ACID | Lipids and lipid-like molecules | Fatty Acyls | placebo - FMT | -0.4298776 | 0.33445534 | 0.22765401 | -0.6201823 | |
| N_ACETYL_D_NORLEUCINE | Organic acids and derivatives | Carboxylic acids and derivatives | placebo - FMT | -0.3956912 | 0.31054202 | 0.23140871 | -0.5708617 | |
| VANILLIN_4_SULFATE | Organic acids and derivatives | Organic sulfuric acids and derivatives | placebo - FMT | 0.72609138 | 0.57261992 | 0.23351865 | 1.04752844 | |
| BETA_RESORCYLIC_ACID | Benzenoids | Benzene and substituted derivatives | placebo - FMT | 0.78277703 | 0.61918904 | 0.23483061 | 1.12930854 | |
| 5__S_METHYL_5__THIOADENOSINE | Undefined | Undefined | placebo - FMT | 0.21175852 | 0.16938949 | 0.23971584 | 0.30550296 | |
| ALPHA_D_XYLOPYRANOSE | Organic oxygen compounds | Organooxygen compounds | placebo - FMT | 0.28677337 | 0.23040127 | 0.24163338 | 0.41372651 | |
| PALMITOYLEICOSAPENTAENOYL_PHOSPHATIDYLCHOLINE | Lipids and lipid-like molecules | Glycerophospholipids | placebo - FMT | -0.3596989 | 0.29041105 | 0.24378514 | -0.5189359 | |
| PRO_LEU | Organic acids and derivatives | Carboxylic acids and derivatives | placebo - FMT | -0.2883372 | 0.23331368 | 0.24476306 | -0.4159827 | |
| 4_HYDROXYCINNAMIC_ACID | Phenylpropanoids and polyketides | Cinnamic acids and derivatives | placebo - FMT | 0.40654741 | 0.32917692 | 0.24504553 | 0.58652393 | |
| PHENOL_SULFATE | Organic acids and derivatives | Organic sulfuric acids and derivatives | placebo - FMT | 1.16225305 | 0.94304819 | 0.24597352 | 1.67677671 | |
| M_HYDROXYBENZOYLECGONINE | Benzenoids | Benzene and substituted derivatives | placebo - FMT | 0.45225633 | 0.3677552 | 0.24692875 | 0.65246796 | |
| N_NAPHTHALEN_2_YL_5_OXOPROLINAMIDE | Organic acids and derivatives | Carboxylic acids and derivatives | placebo - FMT | 0.25356641 | 0.2064961 | 0.24758503 | 0.365819 | |
| OLEOYL_L_CARNITINE | Lipids and lipid-like molecules | Fatty Acyls | placebo - FMT | 0.27710102 | 0.22727776 | 0.25073915 | 0.39977227 | |
| 4___2E__3__3_4_DIHYDROXYPHENYL_PROP_2_ENOYL_OXY_2_3_DIHYDROXY_2_METHYLBUTANOIC_ACID | Undefined | Undefined | placebo - FMT | 0.41857356 | 0.34581944 | 0.25396429 | 0.60387401 | |
| ERYTHRONOLACTONE | Organoheterocyclic compounds | Lactones | placebo - FMT | -0.1030756 | 0.08524099 | 0.25438852 | -0.1487067 | |
| 13_14_DIHYDRO_15_KETOPROSTAGLANDIN_A2 | Lipids and lipid-like molecules | Fatty Acyls | placebo - FMT | 0.42493109 | 0.35233687 | 0.25556261 | 0.61304598 | |
| 3_AMINOCOUMARIN | Phenylpropanoids and polyketides | Coumarins and derivatives | placebo - FMT | 0.29030554 | 0.24273337 | 0.25929053 | 0.41882237 | |
| DEOXYCHOLATE | Lipids and lipid-like molecules | Steroids and steroid derivatives | placebo - FMT | 0.70604174 | 0.59803003 | 0.26507621 | 1.01860292 | |
| NEPSILON_NEPSILON_NEPSILON_TRIMETHYLLYSINE | Organic acids and derivatives | Carboxylic acids and derivatives | placebo - FMT | 0.12468728 | 0.1056687 | 0.2653153 | 0.17988572 | |
| BENZYLDIMETHYLTETRADECYLAMMONIUM_CATION | Benzenoids | Benzene and substituted derivatives | placebo - FMT | 0.23847921 | 0.20246383 | 0.2661132 | 0.34405277 | |
| GLYCYL_L_NORLEUCINE | Organic acids and derivatives | Carboxylic acids and derivatives | placebo - FMT | 0.20542457 | 0.17499388 | 0.26763618 | 0.29636501 | |
| Cys_Val | Organoheterocyclic compounds | Indoles and derivatives | placebo - FMT | 0.30739371 | 0.2641721 | 0.27159379 | 0.44347538 | |
| PHENYLACETYL_L_GLUTAMINE | Organic acids and derivatives | Carboxylic acids and derivatives | placebo - FMT | -0.425169 | 0.36555722 | 0.27180309 | -0.6133892 | |
| 1_2_DIAMINO_2_METHYLPROPANE | Organic nitrogen compounds | Organonitrogen compounds | placebo - FMT | -0.1569045 | 0.1352302 | 0.27288676 | -0.2263654 | |
| GAMMA_GLUTAMYLTYROSINE | Organic acids and derivatives | Carboxylic acids and derivatives | placebo - FMT | 0.54947184 | 0.47984601 | 0.27883379 | 0.79272031 | |
| 1_HEPTADECANOYL_SN_GLYCERO_3_PHOSPHOCHOLINE | Lipids and lipid-like molecules | Glycerophospholipids | placebo - FMT | 0.33579262 | 0.29569778 | 0.28261041 | 0.48444634 | |
| 4_HYDROXY_3_METHYLBENZOIC_ACID | Benzenoids | Benzene and substituted derivatives | placebo - FMT | 0.35180582 | 0.31031806 | 0.28336987 | 0.50754852 | |
| 3_METHOXYPHENCYCLIDINE | Benzenoids | Phenol ethers | placebo - FMT | 0.74152777 | 0.65574737 | 0.28452476 | 1.06979843 | |
| 1H_INDOLE_4_CARBOXALDEHYDE | Organoheterocyclic compounds | Indoles and derivatives | placebo - FMT | 0.35884463 | 0.31854556 | 0.28625668 | 0.51770336 | |
| 3__6_AMINO_9H_PURIN_9_YL_PROPAN_1_OL | Organoheterocyclic compounds | Imidazopyrimidines | placebo - FMT | -0.3073432 | 0.27479121 | 0.28951785 | -0.4434025 | |
| N_L_GAMMA_GLUTAMYL_L_LEUCINE | Organic acids and derivatives | Carboxylic acids and derivatives | placebo - FMT | 0.28295971 | 0.25372191 | 0.29083272 | 0.40822458 | |
| HYPAPHORINE | Organic acids and derivatives | Carboxylic acids and derivatives | placebo - FMT | -1.1936938 | 1.07622586 | 0.29332718 | -1.7221361 | |
| 2__4_MORPHOLINYL__1_PHENYLETHANOL | Organic nitrogen compounds | Organonitrogen compounds | placebo - FMT | -0.2494288 | 0.22515325 | 0.29387449 | -0.3598496 | |
| NAPHTHALENE_2_SULFONIC_ACID | Benzenoids | Naphthalenes | placebo - FMT | 0.11434586 | 0.10405146 | 0.29754901 | 0.1649662 | |
| 13_KETO_9Z_11E_OCTADECADIENOIC_ACID | Lipids and lipid-like molecules | Fatty Acyls | placebo - FMT | -0.1969859 | 0.17945905 | 0.29807754 | -0.2841906 | |
| DIMETHYL_SULFONE | Organosulfur compounds | Sulfonyls | placebo - FMT | -0.8773639 | 0.80263254 | 0.29997919 | -1.2657686 | |
| _DIETHYLAMINO__OXO_ACETIC_ACID | Organic acids and derivatives | Carboxylic acids and derivatives | placebo - FMT | 0.7919506 | 0.72603187 | 0.30094863 | 1.1425432 | |
| N_ACETYL_DL_LEUCINE | Organic acids and derivatives | Carboxylic acids and derivatives | placebo - FMT | 0.29838905 | 0.27369535 | 0.30118767 | 0.4304844 | |
| N_ACETYLLEUCINE | Organic acids and derivatives | Carboxylic acids and derivatives | placebo - FMT | 0.33875953 | 0.31162563 | 0.30251215 | 0.4887267 | |
| NIACINAMIDE | Organoheterocyclic compounds | Pyridines and derivatives | placebo - FMT | 0.35417468 | 0.32902541 | 0.3070158 | 0.51096605 | |
| GLU_PHE | Organic acids and derivatives | Carboxylic acids and derivatives | placebo - FMT | 0.27131699 | 0.25370623 | 0.31001689 | 0.39142768 | |
| 10_HYDROXYDECANOATE | Organic acids and derivatives | Hydroxy acids and derivatives | placebo - FMT | -0.1520489 | 0.14221039 | 0.31011594 | -0.2193603 | |
| CREATININE | Organic acids and derivatives | Carboxylic acids and derivatives | placebo - FMT | -0.1725924 | 0.16144395 | 0.31017122 | -0.2489981 | |
| N__4_CHLOROPHENYL_GLYCINE | Organic acids and derivatives | Carboxylic acids and derivatives | placebo - FMT | 0.22981441 | 0.21509852 | 0.31044582 | 0.33155211 | |
| ASP_LEU | Organic acids and derivatives | Carboxylic acids and derivatives | placebo - FMT | 0.39564017 | 0.37106329 | 0.31138351 | 0.57078812 | |
| 3___1E_3E__HEPTA_1_3_DIENYL_PENTANEDIOIC_ACID | Undefined | Undefined | placebo - FMT | 0.35105886 | 0.32927188 | 0.31141215 | 0.50647088 | |
| PHTHALIC_ACID | Benzenoids | Benzene and substituted derivatives | placebo - FMT | 0.38452047 | 0.36117532 | 0.31207152 | 0.55474577 | |
| NARINGENIN | Phenylpropanoids and polyketides | Flavonoids | placebo - FMT | -0.3709405 | 0.3490304 | 0.31287526 | -0.5351541 | |
| L_2_HYDROXYGLUTARIC_ACID | Organic acids and derivatives | Hydroxy acids and derivatives | placebo - FMT | 0.24958323 | 0.23511881 | 0.31341763 | 0.36007249 | |
| 2____3_5_DICHLOROPHENYL_CARBAMOYL_AMINO_BENZOIC_ACID | Benzenoids | Benzene and substituted derivatives | placebo - FMT | 0.28563761 | 0.26983687 | 0.31470133 | 0.41208797 | |
| DEOXYCARNITINE | Lipids and lipid-like molecules | Fatty Acyls | placebo - FMT | -0.3143862 | 0.29778682 | 0.31592428 | -0.4535634 | |
| ISOQUINOLINE_3_CARBOXYLIC_ACID | Organoheterocyclic compounds | Isoquinolines and derivatives | placebo - FMT | -0.4628146 | 0.44273889 | 0.32047413 | -0.6677003 | |
| 2_HYDROXYCINNAMIC_ACID__PREDOMINANTLY_TRANS | Phenylpropanoids and polyketides | Cinnamic acids and derivatives | placebo - FMT | -0.2695928 | 0.25843741 | 0.32143401 | -0.3889402 | |
| 3_HYDROXYBUTYRYLCARNITINE | Lipids and lipid-like molecules | Fatty Acyls | placebo - FMT | 0.80609556 | 0.783552 | 0.32782798 | 1.16295007 | |
| UMBELLIFERONE_SULFATE | Phenylpropanoids and polyketides | Coumarins and derivatives | placebo - FMT | -1.088232 | 1.06070381 | 0.32909096 | -1.569987 | |
| 8_HYDROXY_5Z_9E_11Z_14Z_EICOSATETRAENOIC_ACID | Lipids and lipid-like molecules | Fatty Acyls | placebo - FMT | 0.32694823 | 0.32219652 | 0.33414849 | 0.4716866 | |
| ISOCITRIC_ACID | Organic acids and derivatives | Carboxylic acids and derivatives | placebo - FMT | 0.24413908 | 0.2416643 | 0.33619921 | 0.35221823 | |
| 1_AMINOCYCLOPROPANECARBOXYLATE | Organic acids and derivatives | Carboxylic acids and derivatives | placebo - FMT | -0.1727293 | 0.17318955 | 0.34211918 | -0.2491957 | |
| LEU_ILE | Organic acids and derivatives | Carboxylic acids and derivatives | placebo - FMT | 0.16749843 | 0.16965353 | 0.3467829 | 0.24164915 | |
| 4_HYDROXYPHENYLLACTIC_ACID | Phenylpropanoids and polyketides | Phenylpropanoic acids | placebo - FMT | -0.2748841 | 0.27952709 | 0.34860941 | -0.396574 | |
| D_LEUCYL_L_ARGININE | Organic acids and derivatives | Carboxylic acids and derivatives | placebo - FMT | -0.1013622 | 0.10331201 | 0.3496706 | -0.1462347 | |
| PALMITATE | Lipids and lipid-like molecules | Fatty Acyls | placebo - FMT | -0.1486721 | 0.15190907 | 0.35081532 | -0.2144885 | |
| TRANS_FERULIC_ACID | Phenylpropanoids and polyketides | Cinnamic acids and derivatives | placebo - FMT | 0.90940672 | 0.93312051 | 0.3527507 | 1.31199657 | |
| 2_AMINOINDAN | Benzenoids | Indanes | placebo - FMT | 0.22574251 | 0.23324965 | 0.35596058 | 0.3256776 | |
| MYRISTOYL_CARNITINE__LC_TDDA__CE20 | Lipids and lipid-like molecules | Fatty Acyls | placebo - FMT | 0.29183219 | 0.30250793 | 0.3574399 | 0.42102485 | |
| URIDINE | Nucleosides, nucleotides, and analogues | Pyrimidine nucleosides | placebo - FMT | -0.3843635 | 0.40000238 | 0.35925887 | -0.5545194 | |
| N_ACETYL_L_GLUTAMINE | Organic acids and derivatives | Carboxylic acids and derivatives | placebo - FMT | 0.22087549 | 0.23088964 | 0.36131 | 0.31865598 | |
| 11_HETE | Lipids and lipid-like molecules | Fatty Acyls | placebo - FMT | 0.34926071 | 0.36627569 | 0.36279396 | 0.50387669 | |
| 3_4_DIHYDROXYPHENYLACETATE | Benzenoids | Phenols | placebo - FMT | -0.209319 | 0.22407426 | 0.37223382 | -0.3019835 | |
| 5_8_11_14_EICOSATETRAENOIC_ACID__16_HYDROXY____5Z_8Z_11Z_14Z_16S__ | Lipids and lipid-like molecules | Fatty Acyls | placebo - FMT | -0.5167168 | 0.55429258 | 0.37318807 | -0.7454647 | |
| 1_OLEOYL_SN_GLYCERO_3_PHOSPHOETHANOLAMINE | Lipids and lipid-like molecules | Glycerophospholipids | placebo - FMT | 0.16690561 | 0.17910203 | 0.37333917 | 0.2407939 | |
| 2_5_DIHYDROXYBENZENESULFONATE | Benzenoids | Benzene and substituted derivatives | placebo - FMT | 0.85459941 | 0.92124659 | 0.3754339 | 1.23292633 | |
| HYPOXANTHINE | Organoheterocyclic compounds | Imidazopyrimidines | placebo - FMT | 0.26815626 | 0.29711388 | 0.38799436 | 0.38686771 | |
| 1_ACETYLINDOLE | Organoheterocyclic compounds | Indoles and derivatives | placebo - FMT | 0.32354665 | 0.36052028 | 0.39057698 | 0.46677915 | |
| _2S__2_AMINO_N_HYDROXY_4__METHYLSULFANYL_BUTANAMIDE | Organic acids and derivatives | Carboxylic acids and derivatives | placebo - FMT | -0.2078431 | 0.23211764 | 0.39160585 | -0.2998541 | |
| 4_HYDROXYPHENETHYL_ALCOHOL | Benzenoids | Phenols | placebo - FMT | 0.19689415 | 0.22005036 | 0.39193826 | 0.28405821 | |
| 4_METHYL_1_2_3_4_TETRAHYDROQUINOLINE | Organoheterocyclic compounds | Quinolines and derivatives | placebo - FMT | 0.36767983 | 0.41692958 | 0.3985459 | 0.53044987 | |
| D_ALANINE | Organic acids and derivatives | Carboxylic acids and derivatives | placebo - FMT | -0.1524847 | 0.17292585 | 0.39858853 | -0.2199889 | |
| 2__4__1_METHYL_1_PHENYLETHYL_PHENOXY_ACETOHYDRAZIDE | Benzenoids | Benzene and substituted derivatives | placebo - FMT | -0.1423912 | 0.16273697 | 0.40211247 | -0.2054271 | |
| OXOPROLINE | Organic acids and derivatives | Carboxylic acids and derivatives | placebo - FMT | 0.07999871 | 0.09167938 | 0.40335099 | 0.11541375 | |
| PHENACETURIC_ACID | Organic acids and derivatives | Carboxylic acids and derivatives | placebo - FMT | -0.2972828 | 0.34492808 | 0.40895141 | -0.4288884 | |
| 1_O_HEXADECYL_2_DEOXY_2_THIO_S_ACETYL_SN_GLYCERYL_3_PHOSPHORYLCHOLINE | Organic nitrogen compounds | Organonitrogen compounds | placebo - FMT | 0.10970653 | 0.12835078 | 0.41270411 | 0.15827307 | |
| 10PHIC10SPC | Benzenoids | Benzene and substituted derivatives | placebo - FMT | -0.4775013 | 0.55971413 | 0.41356197 | -0.6888888 | |
| L_CITRULLINE | Organic acids and derivatives | Carboxylic acids and derivatives | placebo - FMT | 0.18564983 | 0.21840891 | 0.41520669 | 0.2678361 | |
| GLYCOCHOLATE | Lipids and lipid-like molecules | Steroids and steroid derivatives | placebo - FMT | 0.6767518 | 0.7975193 | 0.41597032 | 0.97634647 | |
| D_ARABINOSE | Organic oxygen compounds | Organooxygen compounds | placebo - FMT | 0.20277795 | 0.24051881 | 0.4188895 | 0.29254675 | |
| 2_HYDROXYATRAZINE | Organoheterocyclic compounds | Triazines | placebo - FMT | 0.12584157 | 0.14974215 | 0.42032993 | 0.18155101 | |
| INDOLE_3_ACETAMIDE | Organoheterocyclic compounds | Indoles and derivatives | placebo - FMT | 0.2002802 | 0.24377487 | 0.43046882 | 0.28894325 | |
| TRAMADOL | Benzenoids | Phenol ethers | placebo - FMT | 1.30288215 | 1.59470115 | 0.43295751 | 1.87966162 | |
| GLY_PHE | Organic acids and derivatives | Carboxylic acids and derivatives | placebo - FMT | 0.16427514 | 0.20134325 | 0.43356388 | 0.23699893 | |
| STEARIC_ACID | Lipids and lipid-like molecules | Fatty Acyls | placebo - FMT | 0.05895258 | 0.07274101 | 0.43654679 | 0.08505059 | |
| 1_MYRISTOYL_SN_GLYCERO_3_PHOSPHOCHOLINE | Lipids and lipid-like molecules | Glycerophospholipids | placebo - FMT | 0.21220771 | 0.26357123 | 0.43947119 | 0.306151 | |
| 1_2_DI__9Z_12Z_15Z_OCTADECATRIENOYL__SN_GLYCERO_3_PHOSPHOCHOLINE | Lipids and lipid-like molecules | Glycerophospholipids | placebo - FMT | -0.3223513 | 0.40246131 | 0.44177519 | -0.4650546 | |
| N_PHENYL_3_4_DIHYDRO_2_1H__ISOQUINOLINECARBOTHIOAMIDE | Organoheterocyclic compounds | Tetrahydroisoquinolines | placebo - FMT | -1.1739633 | 1.47358722 | 0.44414507 | -1.693671 | |
| CARNOSINE | Organic acids and derivatives | Peptidomimetics | placebo - FMT | 0.30885573 | 0.38836527 | 0.44492172 | 0.44558463 | |
| 2__3__2_QUINOLYLMETHOXY_ANILINO_BENZOIC_ACID | Undefined | Undefined | placebo - FMT | -0.279885 | 0.35552349 | 0.44939291 | -0.4037887 | |
| L_Valine | Organic acids and derivatives | Carboxylic acids and derivatives | placebo - FMT | -0.1056283 | 0.13455788 | 0.45064926 | -0.1523894 | |
| _2Z__3___AMINOIMINOMETHYL_THIO__2_PROPENOIC_ACID | Lipids and lipid-like molecules | Fatty Acyls | placebo - FMT | 0.86153499 | 1.10341335 | 0.45301218 | 1.24293226 | |
| ___4_HYDROXYPHENYL_ACETYL_AMINO_ACETIC_ACID | Organic acids and derivatives | Carboxylic acids and derivatives | placebo - FMT | -0.2999608 | 0.39306556 | 0.46300773 | -0.4327519 | |
| 7_CHLORO_L_TRYPTOPHAN | Organoheterocyclic compounds | Indoles and derivatives | placebo - FMT | -0.3616003 | 0.47613437 | 0.46510936 | -0.5216789 | |
| OCLACITINIB | Organoheterocyclic compounds | Pyrrolopyrimidines | placebo - FMT | -0.7677324 | 1.01549854 | 0.46707566 | -1.1076037 | |
| URATE | Organoheterocyclic compounds | Imidazopyrimidines | placebo - FMT | -0.7264674 | 0.98008492 | 0.47560092 | -1.0480709 | |
| ISOLEUCINE | Organic acids and derivatives | Carboxylic acids and derivatives | placebo - FMT | 0.23885737 | 0.32518208 | 0.47949316 | 0.34459834 | |
| 1_PALMITOYL_2_HYDROXY_SN_GLYCERO_3_PHOSPHOETHANOLAMINE | Lipids and lipid-like molecules | Glycerophospholipids | placebo - FMT | 0.10906042 | 0.14991196 | 0.48360643 | 0.15734092 | |
| TRIGONELLINE | Alkaloids and derivatives | | placebo - FMT | -0.2373439 | 0.32869813 | 0.48679155 | -0.3424148 | |
| INDOLE_3_METHYL_ACETATE | Organoheterocyclic compounds | Indoles and derivatives | placebo - FMT | 0.4160249 | 0.57708639 | 0.48747838 | 0.60019706 | |
| GLUTARATE | Organic acids and derivatives | Carboxylic acids and derivatives | placebo - FMT | -0.1584062 | 0.21977242 | 0.4875562 | -0.2285318 | |
| N__1H_INDOL_3_YLACETYL_GLYCINE | Organic acids and derivatives | Carboxylic acids and derivatives | placebo - FMT | 0.24121106 | 0.33471128 | 0.48762661 | 0.34799399 | |
| PANTOTHENIC_ACID | Organic oxygen compounds | Organooxygen compounds | placebo - FMT | 0.23978412 | 0.33282564 | 0.48774705 | 0.34593536 | |
| OCTENOYL_CARNITINE__LC_TDDA__CE20 | Lipids and lipid-like molecules | Fatty Acyls | placebo - FMT | 0.36451006 | 0.50727453 | 0.4888578 | 0.52587685 | |
| GLU_GLN | Organic acids and derivatives | Carboxylic acids and derivatives | placebo - FMT | 0.22067074 | 0.31082684 | 0.49395888 | 0.31836058 | |
| PHENYLALANINE | Organic acids and derivatives | Carboxylic acids and derivatives | placebo - FMT | -0.1114066 | 0.15764954 | 0.49590576 | -0.1607258 | |
| LACTIC_ACID | Organic acids and derivatives | Hydroxy acids and derivatives | placebo - FMT | 0.1203332 | 0.17221547 | 0.50064384 | 0.17360411 | |
| ARGININE | Organic acids and derivatives | Carboxylic acids and derivatives | placebo - FMT | -0.0752663 | 0.10842514 | 0.50337788 | -0.1085863 | |
| TAUROCHOLIC_ACID | Lipids and lipid-like molecules | Steroids and steroid derivatives | placebo - FMT | 0.19773027 | 0.28623644 | 0.50541334 | 0.28526448 | |
| 3_CARBOXY_4_METHYL_5_PROPYL_2_FURANPROPIONIC_ACID | Lipids and lipid-like molecules | Fatty Acyls | placebo - FMT | -0.2114603 | 0.30923913 | 0.50963045 | -0.3050728 | |
| TAUROURSODEOXYCHOLIC_ACID | Lipids and lipid-like molecules | Steroids and steroid derivatives | placebo - FMT | 0.34357722 | 0.50266794 | 0.50981277 | 0.49567715 | |
| LAUROYLCARNITINE | Lipids and lipid-like molecules | Fatty Acyls | placebo - FMT | 0.16023606 | 0.23518341 | 0.51113568 | 0.23117177 | |
| AMINOISOBUTANOATE | Organic acids and derivatives | Carboxylic acids and derivatives | placebo - FMT | -0.0920449 | 0.13516904 | 0.51135551 | -0.1327927 | |
| D_ERYTHRO_SPHINGOSINE_1_PHOSPHATE | Lipids and lipid-like molecules | Sphingolipids | placebo - FMT | 0.15621251 | 0.23113404 | 0.51445968 | 0.22536702 | |
| 2__2_QUINOLYL_ETHANOL | Organoheterocyclic compounds | Quinolines and derivatives | placebo - FMT | 0.54252778 | 0.80634355 | 0.51630476 | 0.78270213 | |
| BETAINE | Organic acids and derivatives | Carboxylic acids and derivatives | placebo - FMT | 0.13171557 | 0.19717968 | 0.5192543 | 0.1900254 | |
| 1_PALMITOYL_SN_GLYCERO_3_PHOSPHOCHOLINE | Lipids and lipid-like molecules | Glycerophospholipids | placebo - FMT | 0.03560547 | 0.05363583 | 0.52180462 | 0.05136783 | |
| GABAPENTIN_RELATED_COMPOUND_D | Organoheterocyclic compounds | Azaspirodecane derivatives | placebo - FMT | -0.5436842 | 0.83070781 | 0.52756586 | -0.7843705 | |
| D_ALPHA_CYCLOHEXYLGLYCINE | Organic acids and derivatives | Carboxylic acids and derivatives | placebo - FMT | -0.2725152 | 0.42486223 | 0.53567359 | -0.3931564 | |
| QUINATE | Organic oxygen compounds | Organooxygen compounds | placebo - FMT | 0.45245874 | 0.70567925 | 0.53583107 | 0.65275998 | |
| 5_HYDROXYMETHYLCYTOSINE | Organoheterocyclic compounds | Diazines | placebo - FMT | 0.21037122 | 0.33043115 | 0.53864815 | 0.30350152 | |
| L_PHENYLALANINE__METHYL_ESTER | Organic acids and derivatives | Carboxylic acids and derivatives | placebo - FMT | -0.144982 | 0.22793289 | 0.53901316 | -0.2091648 | |
| N_ACETYL_DL_VALINE | Organic acids and derivatives | Carboxylic acids and derivatives | placebo - FMT | 0.17332177 | 0.27284151 | 0.53953025 | 0.25005046 | |
| PENTAMETHYLENE_BISACETAMIDE | Organic acids and derivatives | Carboxylic acids and derivatives | placebo - FMT | 0.48505042 | 0.76864093 | 0.54216186 | 0.69977984 | |
| PRO_ILE | Organic acids and derivatives | Carboxylic acids and derivatives | placebo - FMT | 0.22807397 | 0.36629444 | 0.54744856 | 0.32904118 | |
| N__4_METHYLPENTANOYL_PHENYLALANINE | Undefined | Undefined | placebo - FMT | -0.1344588 | 0.21747333 | 0.55021454 | -0.193983 | |
| 2_CHLORO_1__4_FLUOROBENZYL_BENZIMIDAZOLE | Organoheterocyclic compounds | Benzimidazoles | placebo - FMT | 0.16972714 | 0.274687 | 0.55045777 | 0.2448645 | |
| 5_HYDROXYMETHYLCYTIDINE | Nucleosides, nucleotides, and analogues | Pyrimidine nucleosides | placebo - FMT | 0.2708286 | 0.43922078 | 0.55126956 | 0.39072308 | |
| N_N_DIMETHYLGUANOSINE | Nucleosides, nucleotides, and analogues | Purine nucleosides | placebo - FMT | 0.12905371 | 0.21138587 | 0.55514296 | 0.18618515 | |
| 3_FURANCARBOXYLIC_ACID | Organoheterocyclic compounds | Furans | placebo - FMT | 0.12293856 | 0.20163581 | 0.55565629 | 0.17736285 | |
| ECGONINE | Alkaloids and derivatives | Tropane alkaloids | placebo - FMT | 0.3370344 | 0.55699003 | 0.55859348 | 0.48623786 | |
| N__ALPHA_LINOLENOYL_TYROSINE | Organic acids and derivatives | Carboxylic acids and derivatives | placebo - FMT | -0.2155074 | 0.35682532 | 0.55932293 | -0.3109114 | |
| HEXANOYL_L_CARNITINE | Lipids and lipid-like molecules | Fatty Acyls | placebo - FMT | 0.18433319 | 0.30561526 | 0.55983635 | 0.26593658 | |
| N_METHYL_L_LEUCINE | Organic acids and derivatives | Carboxylic acids and derivatives | placebo - FMT | -0.2220354 | 0.37634867 | 0.56829972 | -0.3203294 | |
| D_ERYTHRO_N_STEAROYLSPHINGOSINE | Undefined | Undefined | placebo - FMT | 0.16181381 | 0.27834657 | 0.57388081 | 0.23344798 | |
| RIBONIC_ACID | Organic oxygen compounds | Organooxygen compounds | placebo - FMT | 0.1494354 | 0.25719423 | 0.57408681 | 0.2155897 | |
| PROPIONYLCARNITINE | Lipids and lipid-like molecules | Fatty Acyls | placebo - FMT | 0.33123791 | 0.57307883 | 0.57604948 | 0.47787529 | |
| MIDAZOLAM | Organoheterocyclic compounds | Benzodiazepines | placebo - FMT | -0.2545724 | 0.44097652 | 0.57650725 | -0.3672704 | |
| M_TOLUIDINE | Benzenoids | Benzene and substituted derivatives | placebo - FMT | 0.07649654 | 0.13315137 | 0.5783182 | 0.11036117 | |
| METHIONINE | Organic acids and derivatives | Carboxylic acids and derivatives | placebo - FMT | -0.1105815 | 0.19253964 | 0.5784335 | -0.1595353 | |
| N_ALPHA_ACETYL_L_ORNITHINE | Organic acids and derivatives | Carboxylic acids and derivatives | placebo - FMT | 0.25367726 | 0.44453147 | 0.58082459 | 0.36597893 | |
| 13_CIS_RETINOL | Lipids and lipid-like molecules | Prenol lipids | placebo - FMT | 0.34425107 | 0.62072603 | 0.59135915 | 0.49664932 | |
| L_HISTIDINE | Organic acids and derivatives | Carboxylic acids and derivatives | placebo - FMT | -0.0651661 | 0.1177372 | 0.59208827 | -0.0940148 | |
| L_LEUCYL_L_LEUCINE_METHYL_ESTER | Organic acids and derivatives | Carboxylic acids and derivatives | placebo - FMT | 0.18540614 | 0.33517645 | 0.59230452 | 0.26748451 | |
| RIBOFLAVIN | Organoheterocyclic compounds | Pteridines and derivatives | placebo - FMT | 0.1192905 | 0.21592005 | 0.59275591 | 0.17209981 | |
| _2R__3_HYDROXYISOVALEROYLCARNITINE | Undefined | Undefined | placebo - FMT | 0.22009563 | 0.40672833 | 0.60025981 | 0.31753087 | |
| N_ACETYL_METHIONINE | Organic acids and derivatives | Carboxylic acids and derivatives | placebo - FMT | 0.14784843 | 0.27431251 | 0.601694 | 0.2133002 | |
| GUANOSINE | Nucleosides, nucleotides, and analogues | Purine nucleosides | placebo - FMT | 0.16266278 | 0.30330016 | 0.60346943 | 0.23467279 | |
| GAMMA_GLUTAMYLGLUTAMINE | Organic acids and derivatives | Carboxylic acids and derivatives | placebo - FMT | 0.18571072 | 0.34761509 | 0.60484665 | 0.26792393 | |
| CHOLATE | Lipids and lipid-like molecules | Steroids and steroid derivatives | placebo - FMT | 0.31660404 | 0.59342769 | 0.60532999 | 0.45676308 | |
| L_CYSTEINE_GLUTATHIONE_DISULFIDE | Organic acids and derivatives | Carboxylic acids and derivatives | placebo - FMT | 0.30951564 | 0.58238747 | 0.60670293 | 0.44653668 | |
| OLEIC_ACID | Lipids and lipid-like molecules | Fatty Acyls | placebo - FMT | 0.07069917 | 0.13315563 | 0.60704254 | 0.10199735 | |
| 2_ETHYLHYDRACRYLIC_ACID | Lipids and lipid-like molecules | Fatty Acyls | placebo - FMT | 0.15199841 | 0.2868238 | 0.60772056 | 0.21928735 | |
| 3___2S__2_AZETIDINYLMETHOXY_PYRIDINE | Organic oxygen compounds | Organooxygen compounds | placebo - FMT | -0.2541351 | 0.48061768 | 0.60850193 | -0.3666394 | |
| 1_OLEOYL_SN_GLYCERO_3_PHOSPHOCHOLINE | Lipids and lipid-like molecules | Glycerophospholipids | placebo - FMT | 0.06372285 | 0.12109283 | 0.61019912 | 0.09193264 | |
| 7_HYDROXYCOUMARIN_SULFATE | Phenylpropanoids and polyketides | Coumarins and derivatives | placebo - FMT | 0.38190158 | 0.73296476 | 0.61368287 | 0.55096751 | |
| 5BETA_ANDROSTERONE | Lipids and lipid-like molecules | Steroids and steroid derivatives | placebo - FMT | 0.29942937 | 0.57555125 | 0.61421274 | 0.43198526 | |
| 1__3_PYRIDYL__1_BUTANONE_4_CARBOXYLIC_ACID | Undefined | Undefined | placebo - FMT | 0.15598284 | 0.30050424 | 0.61500427 | 0.22503567 | |
| 1__CYCLOHEXYLMETHYL_PROLINE | Organic acids and derivatives | Carboxylic acids and derivatives | placebo - FMT | -0.1176876 | 0.22929403 | 0.61891675 | -0.1697873 | |
| 4_HYDROXYQUINOLINE | Organoheterocyclic compounds | Quinolines and derivatives | placebo - FMT | -0.2492914 | 0.4892829 | 0.62145345 | -0.3596515 | |
| ALPHA_HYODEOXYCHOLIC_ACID_METHYL_ESTER | Undefined | Undefined | placebo - FMT | 0.17166625 | 0.33798753 | 0.62253306 | 0.24766205 | |
| LEUCINE | Organic acids and derivatives | Carboxylic acids and derivatives | placebo - FMT | -0.1156856 | 0.22799899 | 0.62287924 | -0.1668991 | |
| ALANINE | Organic acids and derivatives | Carboxylic acids and derivatives | placebo - FMT | 0.21067454 | 0.41565594 | 0.62324946 | 0.30393912 | |
| 2_METHYLMALONIC_ACID | Organic acids and derivatives | Carboxylic acids and derivatives | placebo - FMT | -0.1076323 | 0.2134029 | 0.62493375 | -0.1552805 | |
| METHADONE | Benzenoids | Benzene and substituted derivatives | placebo - FMT | 0.2576368 | 0.5118151 | 0.62560026 | 0.37169134 | |
| 5_HYDROXYTRYPTOPHOL | Organoheterocyclic compounds | Indoles and derivatives | placebo - FMT | 0.37830213 | 0.75176784 | 0.6257101 | 0.54577461 | |
| 3_INDOLEACETIC_ACID | Organoheterocyclic compounds | Indoles and derivatives | placebo - FMT | 0.15966778 | 0.31886302 | 0.62739028 | 0.23035192 | |
| 2_HYDROXYBUTYRIC_ACID | Organic acids and derivatives | Hydroxy acids and derivatives | placebo - FMT | -0.2055996 | 0.41210823 | 0.62864308 | -0.2966176 | |
| 2_HYDROXYBENZOIC_ACID_SULFATE | Organic acids and derivatives | Organic sulfuric acids and derivatives | placebo - FMT | -0.1477545 | 0.30013028 | 0.63313476 | -0.2131647 | |
| N__4_AMINOBENZOYL__BETA_ALANINE | Organic acids and derivatives | Carboxylic acids and derivatives | placebo - FMT | 0.33090199 | 0.67397269 | 0.63404166 | 0.47739066 | |
| _2__1H_TETRAAZOL_5_YL_PHENOXY_ACETIC_ACID | Organoheterocyclic compounds | Azoles | placebo - FMT | 0.16370652 | 0.34287416 | 0.64330307 | 0.23617858 | |
| 4____3_CHLORO_4_FLUOROPHENYL_SULFONYL_AMINO_BENZOIC_ACID | Benzenoids | Benzene and substituted derivatives | placebo - FMT | 0.06690873 | 0.14090674 | 0.64509833 | 0.09652889 | |
| 4_HYDROXY_3_7_QUINOLINEDICARBOXYLIC_ACID | Organoheterocyclic compounds | Quinolines and derivatives | placebo - FMT | -0.2551057 | 0.53761598 | 0.64532638 | -0.3680397 | |
| CHENODEOXYCHOLIC_ACID | Lipids and lipid-like molecules | Steroids and steroid derivatives | placebo - FMT | 0.44369256 | 0.93891104 | 0.64667102 | 0.64011306 | |
| 3_HYDROXY_2_METHYLBENZOIC_ACID | Benzenoids | Benzene and substituted derivatives | placebo - FMT | 0.14056383 | 0.29853776 | 0.6478568 | 0.20279075 | |
| 2_AMINO_5_METHYLHEXANOIC_ACID | Organic acids and derivatives | Carboxylic acids and derivatives | placebo - FMT | -0.2165996 | 0.4639028 | 0.65057296 | -0.3124872 | |
| ITACONATE | Lipids and lipid-like molecules | Fatty Acyls | placebo - FMT | 0.19899421 | 0.42652406 | 0.65082094 | 0.28708796 | |
| 3_METHYLHISTIDINE__V10_ | Organic acids and derivatives | Carboxylic acids and derivatives | placebo - FMT | -0.1684071 | 0.36599312 | 0.6552601 | -0.2429601 | |
| 3_HYDROXY_3_METHYL_2_3_DIHYDRO_1H_INDOL_2_ONE | Organoheterocyclic compounds | Indoles and derivatives | placebo - FMT | 0.13066496 | 0.28592804 | 0.65744773 | 0.18850969 | |
| THREONINE | Organic acids and derivatives | Carboxylic acids and derivatives | placebo - FMT | -0.0744767 | 0.16502624 | 0.66140063 | -0.1074472 | |
| 6__SIALYL_N_ACETYLLACTOSAMINE | Organic oxygen compounds | Organooxygen compounds | placebo - FMT | -0.2038217 | 0.45378621 | 0.66289515 | -0.2940525 | |
| _3_METHOXYPHENYL_ACETIC_ACID | Benzenoids | Phenol ethers | placebo - FMT | 0.14591876 | 0.33305339 | 0.67060887 | 0.21051627 | |
| DIMETHYL_2_4_BIS_4_HYDROXYPHENYL_CYCLOBUTANE_1_3_DICARBOXYLATE | Undefined | Undefined | placebo - FMT | 0.24120621 | 0.56535247 | 0.67867513 | 0.347987 | |
| 4_BROMO_2_6_DI_TERT_BUTYLPHENOL | Benzenoids | Benzene and substituted derivatives | placebo - FMT | -0.1311951 | 0.31038918 | 0.68147403 | -0.1892745 | |
| ETHYL_2_AMINO_5_METHYL_3_THIOPHENECARBOXYLATE | Organoheterocyclic compounds | Thiophenes | placebo - FMT | 0.2728944 | 0.6600046 | 0.68798699 | 0.39370339 | |
| HYDROXYISOCAPROIC_ACID | Lipids and lipid-like molecules | Fatty Acyls | placebo - FMT | -0.1146046 | 0.27833885 | 0.68921302 | -0.1653394 | |
| INDOLE_3_ACETALDEHYDE_SODIUM_BISULFITE | Organoheterocyclic compounds | Indoles and derivatives | placebo - FMT | 0.14987272 | 0.37102626 | 0.69475926 | 0.21622063 | |
| LONGICAULENONE | Organic oxygen compounds | Organooxygen compounds | placebo - FMT | 0.09070807 | 0.2272995 | 0.69823184 | 0.13086409 | |
| INDOLELACTIC_ACID | Organoheterocyclic compounds | Indoles and derivatives | placebo - FMT | 0.15450944 | 0.39125308 | 0.70120193 | 0.22291001 | |
| ARACHIDONIC_ACID | Lipids and lipid-like molecules | Fatty Acyls | placebo - FMT | -0.0688642 | 0.17469338 | 0.70170854 | -0.0993501 | |
| GUANINE | Organoheterocyclic compounds | Imidazopyrimidines | placebo - FMT | -0.1684079 | 0.43070699 | 0.70399587 | -0.2429612 | |
| 1_METHYL_N_PHENYL_1H_INDOLE_3_CARBOXAMIDE | Benzenoids | Benzene and substituted derivatives | placebo - FMT | 0.0825411 | 0.21121177 | 0.704143 | 0.11908164 | |
| 3_7_12_TRIHYDROXYCHOLAN_24_OIC_ACID__STEREOISOMER_UNKNOWN_ | Undefined | Undefined | placebo - FMT | 0.36717108 | 0.94064518 | 0.7044711 | 0.52971589 | |
| 5__HYDROXYMETHYL__2_FURALDEHYDE | Organic oxygen compounds | Organooxygen compounds | placebo - FMT | -0.0780155 | 0.1998777 | 0.70448783 | -0.1125526 | |
| DEXRAZOXANE | Organic acids and derivatives | Carboxylic acids and derivatives | placebo - FMT | 0.21190607 | 0.5449283 | 0.7055241 | 0.30571584 | |
| N2__2__1H_INDOL_3_YL_ACETYL__L_GLUTAMINE | Organic acids and derivatives | Carboxylic acids and derivatives | placebo - FMT | -0.1354709 | 0.3499435 | 0.70677679 | -0.1954431 | |
| N_ACETYL_PHENYLALANINE | Organic acids and derivatives | Carboxylic acids and derivatives | placebo - FMT | 0.07289549 | 0.18961321 | 0.7086989 | 0.10516596 | |
| 4_HYDROXY_L_ISOLEUCINE | Organic acids and derivatives | Carboxylic acids and derivatives | placebo - FMT | 0.08092457 | 0.21751347 | 0.71762289 | 0.11674948 | |
| 2_8_QUINOLINEDIOL | Organoheterocyclic compounds | Quinolines and derivatives | placebo - FMT | -0.1939027 | 0.52176213 | 0.71792125 | -0.2797425 | |
| MALIC_ACID | Organic acids and derivatives | Hydroxy acids and derivatives | placebo - FMT | -0.2846827 | 0.78051107 | 0.72290156 | -0.4107104 | |
| 1_METHYLNICOTINAMIDE | Organoheterocyclic compounds | Pyridines and derivatives | placebo - FMT | -0.1487916 | 0.40912139 | 0.72366389 | -0.2146609 | |
| 1_6_ANHYDRO_BETA_D_GLUCOSE | Organic acids and derivatives | Carboxylic acids and derivatives | placebo - FMT | -0.0900427 | 0.24944394 | 0.72562841 | -0.1299041 | |
| CREATINE | Organic acids and derivatives | Carboxylic acids and derivatives | placebo - FMT | 0.16647583 | 0.46397381 | 0.72720115 | 0.24017386 | |
| 1_6_ANHYDRO_2_3_O_ISOPROPYLIDENE_BETA_D_MANNOPYRANOSE | Organoheterocyclic compounds | Dioxolopyrans | placebo - FMT | -0.0740393 | 0.20953434 | 0.73116046 | -0.1068161 | |
| 5_METHYLCYTIDINE | Nucleosides, nucleotides, and analogues | Pyrimidine nucleosides | placebo - FMT | -0.1162876 | 0.33012975 | 0.73196282 | -0.1677675 | |
| 1_AMINOCYCLOPENTANECARBOXYLIC_ACID_ETHYL_ESTER | Organic acids and derivatives | Carboxylic acids and derivatives | placebo - FMT | 0.13902133 | 0.3967749 | 0.73332292 | 0.20056538 | |
| 3_INDOLEPROPIONIC_ACID | Organoheterocyclic compounds | Indoles and derivatives | placebo - FMT | -0.2677925 | 0.76458283 | 0.73341883 | -0.3863429 | |
| N_N_DIMETHYLARGININE | Organic acids and derivatives | Carboxylic acids and derivatives | placebo - FMT | 0.03159326 | 0.09097267 | 0.73557674 | 0.04557944 | |
| 5_METHYLCYTOSINE_HYDROCHLORIDE | Organoheterocyclic compounds | Diazines | placebo - FMT | 0.16712637 | 0.49066171 | 0.74044139 | 0.24111239 | |
| N__3_CHLOROPHENYL__3_HYDROXY_2_NAPHTHAMIDE | Benzenoids | Naphthalenes | placebo - FMT | 0.33647819 | 0.9956756 | 0.74239588 | 0.48543542 | |
| CROTONIC_ACID | Lipids and lipid-like molecules | Fatty Acyls | placebo - FMT | 0.06451544 | 0.19349397 | 0.7457003 | 0.0930761 | |
| KYNURENIC_ACID | Organoheterocyclic compounds | Quinolines and derivatives | placebo - FMT | -0.1767595 | 0.53231971 | 0.74670266 | -0.25501 | |
| AMINOADIPATE | Organic acids and derivatives | Carboxylic acids and derivatives | placebo - FMT | 0.09780334 | 0.30122773 | 0.75211156 | 0.14110039 | |
| DECANOIC_ACID | Lipids and lipid-like molecules | Fatty Acyls | placebo - FMT | 0.33983611 | 1.06069579 | 0.75526728 | 0.49027987 | |
| GLU_THR | Organic acids and derivatives | Carboxylic acids and derivatives | placebo - FMT | 0.06281803 | 0.1995278 | 0.75935905 | 0.09062726 | |
| 4__2____3S__2_3_DIHYDROXY_2___1S__1_HYDROXYETHYL_BUTANOYL_OXYMETHYL_ANILINO__4_OXOBUTANOIC_ACID | Benzenoids | Benzene and substituted derivatives | placebo - FMT | 0.06797056 | 0.21653028 | 0.76004145 | 0.09806079 | |
| L_CARNITINE | Organic nitrogen compounds | Organonitrogen compounds | placebo - FMT | 0.03795712 | 0.12123229 | 0.76064156 | 0.05476054 | |
| TROPIC_ACID | Organic acids and derivatives | Hydroxy acids and derivatives | placebo - FMT | -0.0651243 | 0.2140018 | 0.76712505 | -0.0939545 | |
| GLYCEROPHOSPHOCHOLINE | Lipids and lipid-like molecules | Glycerophospholipids | placebo - FMT | 0.04103882 | 0.13512482 | 0.7675736 | 0.0592065 | |
| N_ACETYLNEURAMINIC_ACID | Organic oxygen compounds | Organooxygen compounds | placebo - FMT | -0.1289856 | 0.42508167 | 0.7677758 | -0.1860869 | |
| N_FORMYLMETHIONINE | Organic acids and derivatives | Carboxylic acids and derivatives | placebo - FMT | -0.0813522 | 0.26811042 | 0.76778267 | -0.1173664 | |
| OCTANOYLCARNITINE | Lipids and lipid-like molecules | Fatty Acyls | placebo - FMT | 0.08807472 | 0.29098052 | 0.76833427 | 0.12706496 | |
| LEWIS_X_TRISACCHARIDE | Organic oxygen compounds | Organooxygen compounds | placebo - FMT | -0.1397334 | 0.46460185 | 0.76975812 | -0.2015926 | |
| 3_HYDROXYISOVALERIC_ACID | Lipids and lipid-like molecules | Fatty Acyls | placebo - FMT | -0.1327167 | 0.44406073 | 0.77115749 | -0.1914697 | |
| 1_STEAROYL_2_HYDROXY_SN_GLYCERO_3_PHOSPHOCHOLINE | Lipids and lipid-like molecules | Glycerophospholipids | placebo - FMT | -0.0358075 | 0.12411308 | 0.77885062 | -0.0516592 | |
| 2_HYDROXYHEXADECANOIC_ACID | Lipids and lipid-like molecules | Fatty Acyls | placebo - FMT | 0.04078819 | 0.14406335 | 0.78285416 | 0.05884492 | |
| METHIONINE_SULFONE__AIF__CE10__MS2DEC | Organic acids and derivatives | Carboxylic acids and derivatives | placebo - FMT | 0.18139587 | 0.66441625 | 0.79039657 | 0.26169892 | |
| TRIMETHYLAMINE_N_OXIDE | Organic nitrogen compounds | Organonitrogen compounds | placebo - FMT | -0.0750475 | 0.27519437 | 0.79062668 | -0.1082707 | |
| 2_HYDROXYBENZALDEHYDE | Organic oxygen compounds | Organooxygen compounds | placebo - FMT | 0.06693493 | 0.24960387 | 0.79402303 | 0.0965667 | |
| ECTOINE | Organic acids and derivatives | Carboxylic acids and derivatives | placebo - FMT | -0.1019697 | 0.39917919 | 0.80355412 | -0.1471111 | |
| METHIONINESULFOXIDE | Organic acids and derivatives | Carboxylic acids and derivatives | placebo - FMT | 0.16189329 | 0.6395617 | 0.80529423 | 0.23356265 | |
| 2_ACETAMIDO_3__4_HYDROXY_3_METHOXYPHENYL_PROPANOIC_ACID | Organic acids and derivatives | Carboxylic acids and derivatives | placebo - FMT | -0.1297478 | 0.51510053 | 0.80622857 | -0.1871865 | |
| 1__1Z_OCTADECENYL__SN_GLYCERO_3_PHOSPHOCHOLINE | Lipids and lipid-like molecules | Glycerophospholipids | placebo - FMT | 0.05214373 | 0.20834867 | 0.80744406 | 0.0752275 | |
| 1_O_OCTYL_2_O__N_METHYLCARBAMOYL__SN_GLYCERYL_3_PHOSPHORYLCHOLINE | Lipids and lipid-like molecules | Glycerophospholipids | placebo - FMT | -0.0273332 | 0.11014619 | 0.80903675 | -0.0394335 | |
| 1_3_DIHYDRO__2H__INDOL_2_ONE | Organoheterocyclic compounds | Indoles and derivatives | placebo - FMT | 0.12025005 | 0.48535231 | 0.8093345 | 0.17348416 | |
| 1_O_HEXADECYL_2_O_ACETYL_SN_GLYCERYL_3_PHOSPHORYLCHOLINE | Lipids and lipid-like molecules | Glycerophospholipids | placebo - FMT | 0.05103202 | 0.20911885 | 0.81213859 | 0.07362364 | |
| _5AR_10AR__OCTAHYDRODIPYRROLO_1_2_A:1__2__D_PYRAZINE_5_10_DIONE | Organic acids and derivatives | Carboxylic acids and derivatives | placebo - FMT | 0.10918812 | 0.44869319 | 0.81265581 | 0.15752517 | |
| 3_HYDROXY_2___9Z_12Z__OCTADECA_9_12_DIENOYLOXY_PROPYL_2__TRIMETHYLAZANIUMYL_ETHYL_PHOSPHATE | Lipids and lipid-like molecules | Glycerophospholipids | placebo - FMT | -0.0476643 | 0.19875761 | 0.81532079 | -0.068765 | |
| N_ACETYL_BETA_ALANINE | Organic acids and derivatives | Carboxylic acids and derivatives | placebo - FMT | 0.04635519 | 0.19642305 | 0.81819852 | 0.0668764 | |
| CARPROFEN | Organoheterocyclic compounds | Indoles and derivatives | placebo - FMT | -0.1891818 | 0.80177917 | 0.81823188 | -0.2729316 | |
| 2_AMINO_3_PHENYLBUTANOIC_ACID | Phenylpropanoids and polyketides | Phenylpropanoic acids | placebo - FMT | -0.0542851 | 0.23653539 | 0.8231057 | -0.0783168 | |
| 2_4_DIHYDROXYBUTANOIC_ACID | Organic acids and derivatives | Hydroxy acids and derivatives | placebo - FMT | 0.04121276 | 0.18074736 | 0.82423073 | 0.05945744 | |
| 9_HODE | Lipids and lipid-like molecules | Fatty Acyls | placebo - FMT | -0.0428806 | 0.19288715 | 0.82854792 | -0.0618636 | |
| 2_ACETAMIDO_2_DEOXY_3_O__BETA_D_GALACTOPYRANOSYL__D_GLUCOPYRANOSE | Organic oxygen compounds | Organooxygen compounds | placebo - FMT | -0.0662464 | 0.30932384 | 0.83472203 | -0.0955734 | |
| N2_ACETYLORNITHINE | Organic acids and derivatives | Carboxylic acids and derivatives | placebo - FMT | 0.08026297 | 0.38243758 | 0.83798168 | 0.11579498 | |
| SYRINGIC_ACID | Benzenoids | Benzene and substituted derivatives | placebo - FMT | 0.03881804 | 0.1895083 | 0.84180975 | 0.0560026 | |
| _2_OXOBENZO_CD_INDOL_1_2H__YL_ACETIC_ACID | Organoheterocyclic compounds | Isoindoles and derivatives | placebo - FMT | -0.2344379 | 1.18798371 | 0.8475141 | -0.3382224 | |
| 2_KETOHEXANOIC_ACID | Organic acids and derivatives | Keto acids and derivatives | placebo - FMT | 0.04092905 | 0.20985578 | 0.84927178 | 0.05904814 | |
| 8_11_TRIDECADIENOIC_ACID__13__3_PENTYL_2_OXIRANYL_____8Z_11Z__ | Lipids and lipid-like molecules | Fatty Acyls | placebo - FMT | 0.0236685 | 0.12157996 | 0.84954605 | 0.03414643 | |
| 2__1H_PYRROL_1_YL_BENZOIC_ACID | Organoheterocyclic compounds | Pyrroles | placebo - FMT | -0.1698537 | 0.91674379 | 0.85671394 | -0.245047 | |
| GAMMA_GLUTAMYLMETHIONINE | Organic acids and derivatives | Carboxylic acids and derivatives | placebo - FMT | -0.1074427 | 0.58173745 | 0.85716195 | -0.155007 | |
| L_GLUTAMINE | Organic acids and derivatives | Carboxylic acids and derivatives | placebo - FMT | -0.0397682 | 0.21594844 | 0.85757204 | -0.0573733 | |
| 1_STEAROYL_2_HYDROXY_SN_GLYCERO_3_PHOSPHOETHANOLAMINE | Lipids and lipid-like molecules | Glycerophospholipids | placebo - FMT | 0.03442804 | 0.18779693 | 0.85820604 | 0.04966916 | |
| VALINE | Organic acids and derivatives | Carboxylic acids and derivatives | placebo - FMT | -0.1152707 | 0.63195365 | 0.85891062 | -0.1663005 | |
| 1_LINOLEOYL_SN_GLYCERO_3_PHOSPHORYLCHOLINE | Lipids and lipid-like molecules | Glycerophospholipids | placebo - FMT | 0.01954329 | 0.10753697 | 0.85942123 | 0.02819501 | |
| LINOLEOYLCARNITINE | Lipids and lipid-like molecules | Fatty Acyls | placebo - FMT | 0.04607339 | 0.25485316 | 0.86014846 | 0.06646985 | |
| D_L_N_N_DIDESMETHYL_O_DESMETHYLVENLAFAXINE | Organic oxygen compounds | Organooxygen compounds | placebo - FMT | 0.14400399 | 0.80703848 | 0.86194437 | 0.20775384 | |
| OXOGLUTARATE | Organic acids and derivatives | Keto acids and derivatives | placebo - FMT | 0.03393834 | 0.19140237 | 0.86280171 | 0.04896268 | |
| 5_METHYLCYTOSINE | Organoheterocyclic compounds | Diazines | placebo - FMT | 0.03838979 | 0.22090462 | 0.86550245 | 0.05538476 | |
| _2E__3__1H_INDOL_2_YL__2_PROPENOIC_ACID | Organoheterocyclic compounds | Indoles and derivatives | placebo - FMT | 0.12011739 | 0.70745788 | 0.86856307 | 0.17329276 | |
| L_TYROSINE | Organic acids and derivatives | Carboxylic acids and derivatives | placebo - FMT | 0.1275602 | 0.76056143 | 0.87014811 | 0.18403046 | |
| GLY_VAL | Organic acids and derivatives | Carboxylic acids and derivatives | placebo - FMT | -0.0394593 | 0.23740723 | 0.87130456 | -0.0569278 | |
| 3_PHENYLLACTIC_ACID | Phenylpropanoids and polyketides | Phenylpropanoic acids | placebo - FMT | 0.05706396 | 0.35088229 | 0.8740492 | 0.08232589 | |
| PROLINE | Organic acids and derivatives | Carboxylic acids and derivatives | placebo - FMT | 0.03440283 | 0.21415006 | 0.8755694 | 0.0496328 | |
| _2E__4_ANILINO_4_OXO_2_BUTENOIC_ACID | Benzenoids | Benzene and substituted derivatives | placebo - FMT | 0.0598589 | 0.39751965 | 0.88330035 | 0.08635814 | |
| 7_KETODEOXYCHOLATE | Lipids and lipid-like molecules | Steroids and steroid derivatives | placebo - FMT | -0.1168588 | 0.77630803 | 0.8833384 | -0.1685916 | |
| SERINE | Organic acids and derivatives | Carboxylic acids and derivatives | placebo - FMT | -0.0472463 | 0.32251239 | 0.88644215 | -0.068162 | |
| _3_4_DIHYDROXYPHENYL_ETHANOL | Undefined | Undefined | placebo - FMT | 0.07253895 | 0.54550734 | 0.89685078 | 0.10465158 | |
| N__PIPERIDIN_4_YL_METHANESULFONAMIDE | Organoheterocyclic compounds | Piperidines | placebo - FMT | -0.1037919 | 0.83640286 | 0.90370046 | -0.14974 | |
| 3_METHYL_2_OXOVALERATE | Organic acids and derivatives | Keto acids and derivatives | placebo - FMT | 0.02897398 | 0.2392925 | 0.90602463 | 0.04180061 | |
| 2_HYDROXY_2_METHYLBUTYRIC_ACID | Lipids and lipid-like molecules | Fatty Acyls | placebo - FMT | -0.0279412 | 0.23299295 | 0.90691945 | -0.0403106 | |
| ISOVALEROPHENONE | Organic oxygen compounds | Organooxygen compounds | placebo - FMT | -0.0208917 | 0.17509194 | 0.90738601 | -0.0301404 | |
| _4_METHYLPHENYL_OXIDANESULFONIC_ACID | Organic acids and derivatives | Organic sulfuric acids and derivatives | placebo - FMT | -0.0141372 | 0.1226274 | 0.91050072 | -0.0203957 | |
| CYTOSINE | Organoheterocyclic compounds | Diazines | placebo - FMT | 0.0285119 | 0.26111253 | 0.91520879 | 0.04113398 | |
| PROPOFOL_BETA_D_GLUCURONIDE | Organic oxygen compounds | Organooxygen compounds | placebo - FMT | -0.0286358 | 0.26996802 | 0.91762367 | -0.0413127 | |
| _1_METHYL_1H_IMIDAZOL_4_YL_ACETIC_ACID | Organoheterocyclic compounds | Azoles | placebo - FMT | -0.0201916 | 0.19046635 | 0.91766985 | -0.0291303 | |
| METHYL__9Z_14Z__12_13_16_TRIHYDROXYOCTADECA_9_14_DIENOATE | Lipids and lipid-like molecules | Fatty Acyls | placebo - FMT | -0.011386 | 0.11227198 | 0.92122624 | -0.0164265 | |
| TYR_LEU | Organic acids and derivatives | Carboxylic acids and derivatives | placebo - FMT | 0.04968356 | 0.50579846 | 0.9236923 | 0.07167823 | |
| 2__HYDROXYMETHYL__5_METHYL_4H_7H__1_2_4_TRIAZOLO_1_5_A_PYRIMIDIN_7_ONE | Organoheterocyclic compounds | Triazolopyrimidines | placebo - FMT | -0.0174376 | 0.17859588 | 0.92414954 | -0.0251572 | |
| ONDANSETRON | Organoheterocyclic compounds | Indoles and derivatives | placebo - FMT | -0.0180023 | 0.18923385 | 0.92608867 | -0.0259719 | |
| 12_HYDROXYOCTADECANOIC_ACID | Lipids and lipid-like molecules | Fatty Acyls | placebo - FMT | 0.02525895 | 0.27680084 | 0.9290935 | 0.03644096 | |
| TAURINE | Organic acids and derivatives | Organic sulfonic acids and derivatives | placebo - FMT | 0.02922998 | 0.32210835 | 0.92948654 | 0.04216995 | |
| 3_HYDROXYISOBUTYRIC_ACID | Organic acids and derivatives | Hydroxy acids and derivatives | placebo - FMT | 0.03321686 | 0.36840583 | 0.92993744 | 0.0479218 | |
| 5__2_FURYL__4H_1_2_4_TRIAZOL_3_AMINE | Organoheterocyclic compounds | Azoles | placebo - FMT | -0.0293847 | 0.34196213 | 0.93321846 | -0.0423932 | |
| PANTOTHENATE | Organic acids and derivatives | Carboxylic acids and derivatives | placebo - FMT | 0.02329544 | 0.27289929 | 0.93365788 | 0.03360822 | |
| N_ACETYL_O_FLUORO_DL_PHENYLALANINE | Organic acids and derivatives | Carboxylic acids and derivatives | placebo - FMT | -0.0623411 | 0.76153863 | 0.93637171 | -0.0899393 | |
| METHYL__4AR__5_6_DIHYDROXY_1_1_DIMETHYL_7_PROPAN_2_YL_2_3_4_9_10_10A_HEXAHYDROPHENANTHRENE_4A_CARBOXYLATE | Lipids and lipid-like molecules | Prenol lipids | placebo - FMT | -0.0830928 | 1.03468483 | 0.93757729 | -0.1198775 | |
| 3_INDOXYLSULFATE_POTASSIUM_SALT | Organic acids and derivatives | Organic sulfuric acids and derivatives | placebo - FMT | 0.04163427 | 0.57819951 | 0.94401635 | 0.06006556 | |
| 1_METHYL_2_3_4_9_TETRAHYDRO_1H_BETA_CARBOLINE_1_CARBOXYLIC_ACID | Alkaloids and derivatives | Harmala alkaloids | placebo - FMT | -0.0437352 | 0.75259357 | 0.9548037 | -0.0630966 | |
| 1_DECANOYL_2_HYDROXY_SN_GLYCERO_3_PHOSPHOCHOLINE | Lipids and lipid-like molecules | Glycerophospholipids | placebo - FMT | -0.0079623 | 0.15773603 | 0.96073485 | -0.0114872 | |
| 2_6_DIMETHYLISONICOTINIC_ACID | Organoheterocyclic compounds | Pyridines and derivatives | placebo - FMT | 0.01088199 | 0.22397486 | 0.96220609 | 0.0156994 | |
| 1_HEXADECYL_SN_GLYCERO_3_PHOSPHOCHOLINE | Lipids and lipid-like molecules | Glycerophospholipids | placebo - FMT | 0.01136382 | 0.25515908 | 0.96535378 | 0.01639452 | |
| CORTISOL | Lipids and lipid-like molecules | Steroids and steroid derivatives | placebo - FMT | -0.0145816 | 0.34076379 | 0.96671058 | -0.0210368 | |
| INOSINE | Nucleosides, nucleotides, and analogues | Purine nucleosides | placebo - FMT | -0.026204 | 0.80761358 | 0.97475471 | -0.0378043 | |
| TRANS_TRAUMATIC_ACID | Lipids and lipid-like molecules | Fatty Acyls | placebo - FMT | -0.0073476 | 0.38159549 | 0.98501647 | -0.0106004 | |
| N_METHYL_L_ASPARAGINE | Organic acids and derivatives | Carboxylic acids and derivatives | placebo - FMT | -0.0021809 | 0.11956843 | 0.98580636 | -0.0031464 | |

Log2FC values are provided for descriptive context; statistical inference is based on longitudinal mixed-effects model estimates.

**Table 4.** GCMS Metabolite FgseaRes

| pathway | SuperPathway | pval | padj | log2err | ES | NES | leadingEdge |
| --- | --- | --- | --- | --- | --- | --- | --- |
| Phenols | Benzenoids | 0.013286864 | 0.198044573 | 0.380730401 | 0.950570944 | 1.423277665 | c("3_ETHYLPHENOL", "3_4_5_TRIMETHOXYPHENOL", "4_HYDROXYPHENETHYL_ALCOHOL") |
| Organooxygen compounds | Organic oxygen compounds | 0.013658246 | 0.198044573 | 0.380730401 | 0.784248246 | 1.582700959 | c("RAC_N_N_DIDESMETHYL_O_DESMETHYLVENLAFAXINE_GLUCURONIDE", "RAC__1R_3AR_6AS__OCTAHYDROCYCLOPENTA_C_PYRROLE_1_CARBOXYLIC_ACID", "SUCROSE", "N_ACETYLNEURAMINATE", "GLYCERATE", "2_AMINOACETOPHENONE", "L_KYNURENINE") |
| Keto acids and derivatives | Organic acids and derivatives | 0.039160839 | 0.34673913 | 0.271288555 | 0.925871282 | 1.386295178 | c("4__2_FURYL__2_4_DIOXOBUTANOIC_ACID", "2_KETOHEXANOIC_ACID") |
| Organonitrogen compounds | Organic nitrogen compounds | 0.048913043 | 0.34673913 | 0.237793834 | 0.825653634 | 1.384476561 | c("METHACHOLINE_CATION", "CHOLINE_CHLORIDE", "SPERMIDINE") |
| Organic sulfuric acids and derivatives | Organic acids and derivatives | 0.059782609 | 0.34673913 | 0.213927855 | 0.809933387 | 1.358116459 | c("PHENOL_SULFATE", "ETHYL_SULFATE", "VANILLIN_4_SULFATE", "N_OCTYL_SULFATE", "C12_AS__TENTATIVE_") |
| Benzene and substituted derivatives | Benzenoids | 0.112607099 | 0.4984375 | 0.143758989 | 0.662112507 | 1.324571175 | c("5__ACETYLAMINO__2_HYDROXYBENZOIC_ACID", "N__4_BROMOPHENYL_ACRYLAMIDE", "BETA_RESORCYLIC_ACID", "2_5_DIHYDROXYBENZENESULFONATE", "N__4_CHLOROBENZOYL_TRYPTOPHAN", "PROPYLPARABEN", "M_HYDROXYBENZOYLECGONINE", "ANILINE", "PHTHALIC_ACID", "4_HYDROXY_3_METHYLBENZOIC_ACID") |
| Carboxylic acids and derivatives | Organic acids and derivatives | 0.126845805 | 0.4984375 | 0.249246554 | -0.325722371 | -1.130613608 | c("HOMOARGININE", "2_PIPERIDINECARBOXAMIDE", "1_ACETAMIDOCYCLOPENTANE_1_CARBOXYLIC_ACID", "GABAPENTIN", "LYSINE", "HYPAPHORINE", "5_AMINOPENTANAMIDE", "PIPECOLATE", "N_ACETYL_D_NORLEUCINE", "PHENYLACETYL_L_GLUTAMINE") |
| Phenol ethers | Benzenoids | 0.1375 | 0.4984375 | 0.138022242 | 0.803368274 | 1.25341228 | c("TRAMADOL_N_OXIDE", "TRAMADOL", "3_METHOXYPHENCYCLIDINE", "GEMFIBROZIL") |
| Piperidines | Organoheterocyclic compounds | 0.222222222 | 0.623140496 | 0.114266502 | 0.858796475 | 1.187806581 | c("N__3_PIPERIDINYL_ACETAMIDE", "N__PIPERIDIN_4_YL_METHANESULFONAMIDE") |
| Phenylpropanoic acids | Phenylpropanoids and polyketides | 0.223214286 | 0.623140496 | 0.160801401 | -0.845617684 | -1.294822409 | 4_HYDROXYPHENYLLACTIC_ACID |
| Azoles | Organoheterocyclic compounds | 0.236363636 | 0.623140496 | 0.101350744 | 0.794028434 | 1.188888576 | c("ALLANTOIN", "_2__1H_TETRAAZOL_5_YL_PHENOXY_ACETIC_ACID") |
| Quinolines and derivatives | Organoheterocyclic compounds | 0.422535211 | 0.85014881 | 0.123842171 | -0.523060263 | -1.006034087 | ETHYL_4_HYDROXYQUINOLINE_2_CARBOXYLATE |
| Undefined | Undefined | 0.449293967 | 0.85014881 | 0.062249043 | 0.553138903 | 1.04085188 | c("5_METHOXYSALICYLIC_ACID_SULFATE", "3_HYDROXY_3___4_7_7_TRIMETHYL_3_BICYCLO_221_HEPTANYL_OXYCARBONYL_PENTANEDIOIC_ACID", "4___2E__3__3_4_DIHYDROXYPHENYL_PROP_2_ENOYL_OXY_2_3_DIHYDROXY_2_METHYLBUTANOIC_ACID", "3___1E_3E__HEPTA_1_3_DIENYL_PENTANEDIOIC_ACID", "DIETHYL_2___2_HYDROXYANILINO_METHYLENE_MALONATE", "5__S_METHYL_5__THIOADENOSINE") |
| Naphthalenes | Benzenoids | 0.46993007 | 0.85014881 | 0.064214088 | 0.683061807 | 1.02273967 | c("4_METHOXYNAPHTHALEN_1_AMINE", "2_NAPHTHYLAMINE", "NAPHTHALENE_2_SULFONIC_ACID", "N__3_CHLOROPHENYL__3_HYDROXY_2_NAPHTHAMIDE") |
| Steroids and steroid derivatives | Lipids and lipid-like molecules | 0.488559892 | 0.85014881 | 0.060509299 | 0.602137185 | 1.023679879 | c("DEOXYCHOLATE", "GLYCOCHOLATE", "TAUROURSODEOXYCHOLIC_ACID", "CHENODEOXYCHOLIC_ACID", "CHOLATE", "5BETA_ANDROSTERONE", "TAUROCHOLIC_ACID") |
| Coumarins and derivatives | Phenylpropanoids and polyketides | 0.50297619 | 0.85014881 | 0.101350744 | -0.653379348 | -1.000464203 | UMBELLIFERONE_SULFATE |
| Indoles and derivatives | Organoheterocyclic compounds | 0.533742331 | 0.85014881 | 0.052057003 | 0.491639752 | 0.961297744 | c("L_TRYPTOPHAN", "INDOLE", "3_FORMYLINDOLE", "1H_INDOLE_4_CARBOXALDEHYDE", "Cys_Val", "1_ACETYLINDOLE", "INDOLE_3_METHYL_ACETATE", "5_HYDROXYTRYPTOPHOL", "INDOLE_3_ACETAMIDE") |
| Glycerophospholipids | Lipids and lipid-like molecules | 0.564102564 | 0.85014881 | 0.130105631 | -0.368922398 | -0.920832483 | c("PALMITOYLEICOSAPENTAENOYL_PHOSPHATIDYLCHOLINE", "1__1Z_HEXADECENYL__SN_GLYCERO_3_PHOSPHOCHOLINE", "1_2_DI__9Z_12Z_15Z_OCTADECATRIENOYL__SN_GLYCERO_3_PHOSPHOCHOLINE") |
| Pyridines and derivatives | Organoheterocyclic compounds | 0.572072072 | 0.85014881 | 0.058221624 | 0.667211729 | 0.964556423 | c("NIACINAMIDE", "2_6_DIMETHYLISONICOTINIC_ACID") |
| Pyrimidine nucleosides | Nucleosides, nucleotides, and analogues | 0.586309524 | 0.85014881 | 0.092259726 | -0.588606474 | -0.901283012 | URIDINE |
| Cinnamic acids and derivatives | Phenylpropanoids and polyketides | 0.648648649 | 0.895400126 | 0.052375908 | 0.634425561 | 0.917159011 | c("TRANS_FERULIC_ACID", "4_HYDROXYCINNAMIC_ACID") |
| Prenol lipids | Lipids and lipid-like molecules | 0.70531401 | 0.895400126 | 0.051740546 | 0.627646661 | 0.86810188 | c("13_CIS_RETINOL", "METHYL__4AR__5_6_DIHYDROXY_1_1_DIMETHYL_7_PROPAN_2_YL_2_3_4_9_10_10A_HEXAHYDROPHENANTHRENE_4A_CARBOXYLATE") |
| Benzodiazepines | Organoheterocyclic compounds | 0.710144928 | 0.895400126 | 0.051426492 | 0.623390422 | 0.862215051 | c("_3R__5__2_FLUOROPHENYL__3__1H_INDOL_3_YLMETHYL__1_METHYL_3H_1_4_BENZODIAZEPIN_2_ONE", "MIDAZOLAM") |
| Purine nucleosides | Nucleosides, nucleotides, and analogues | 0.837837838 | 0.960969044 | 0.040657934 | 0.527913776 | 0.763179963 | c("GUANOSINE", "N_N_DIMETHYLGUANOSINE", "INOSINE") |
| Imidazopyrimidines | Organoheterocyclic compounds | 0.864130435 | 0.960969044 | 0.034566432 | 0.421499618 | 0.70678105 | c("XANTHINE", "PARAXANTHINE", "CAFFEINE", "HYPOXANTHINE") |
