# Supplementary material for: Serum metabolomics of diabetic dogs treated with daily administration of a commercially available lyophilized feces preparation
Source: Vet Res Commun. 2026 Mar 27;50(3):229. doi: 10.1007/s11259-026-11181-9 (PMC13031211; doi:10.1007/s11259-026-11181-9)
Supplement: Supplementary file 8 — Supplementary Material 8. [file 11259_2026_11181_MOESM8_ESM.docx]

**METHODS**

**Study Design**

This prospective, randomized, placebo-controlled, double-blinded clinical trial (IACUC#19235) was conducted at the University of Illinois between September 2021 and June 2022. Despite targeting a sample size of 24 dogs based on a priori sample size power estimation (see the power sample size estimation below), we were able to enroll only 12 diabetic patients due to recruitment constraints. The 12 diabetic dogs were enrolled from public sources and local referral clinics after obtaining written informed consent from their owners. Dogs were randomly assigned to receive either a placebo or LFP treatment using an online randomization tool (http://www.randomizer.org).

Eligible dogs had a confirmed diagnosis of stable DM and had received insulin therapy for at least 14 days before enrollment. Additional inclusion criteria included maintaining stable body weight (±5% of baseline) for 14 days prior to enrollment, absence of obvious diabetic clinical signs (e.g., excessive thirst, urination, hunger, or weight loss), and no exposure to steroids, antibiotics, or probiotics for at least 14 days before and throughout the study. Dietary regimens remained unchanged during the study.

Clinical evaluations occurred at baseline and at 2, 4, 6, and 8 weeks. Evaluations included a physical examination, complete blood count, serum biochemical profile, and urinalysis. The CBC, serum biochemical profiles, and urinalysis were part of routine clinical monitoring to ensure patient stability and detect adverse events, rather than as predefined efficacy endpoints. These data were not used for hypothesis testing. Serum, urine, and fecal samples were collected at each visit and stored at -80°C until analysis (Fig. 1).


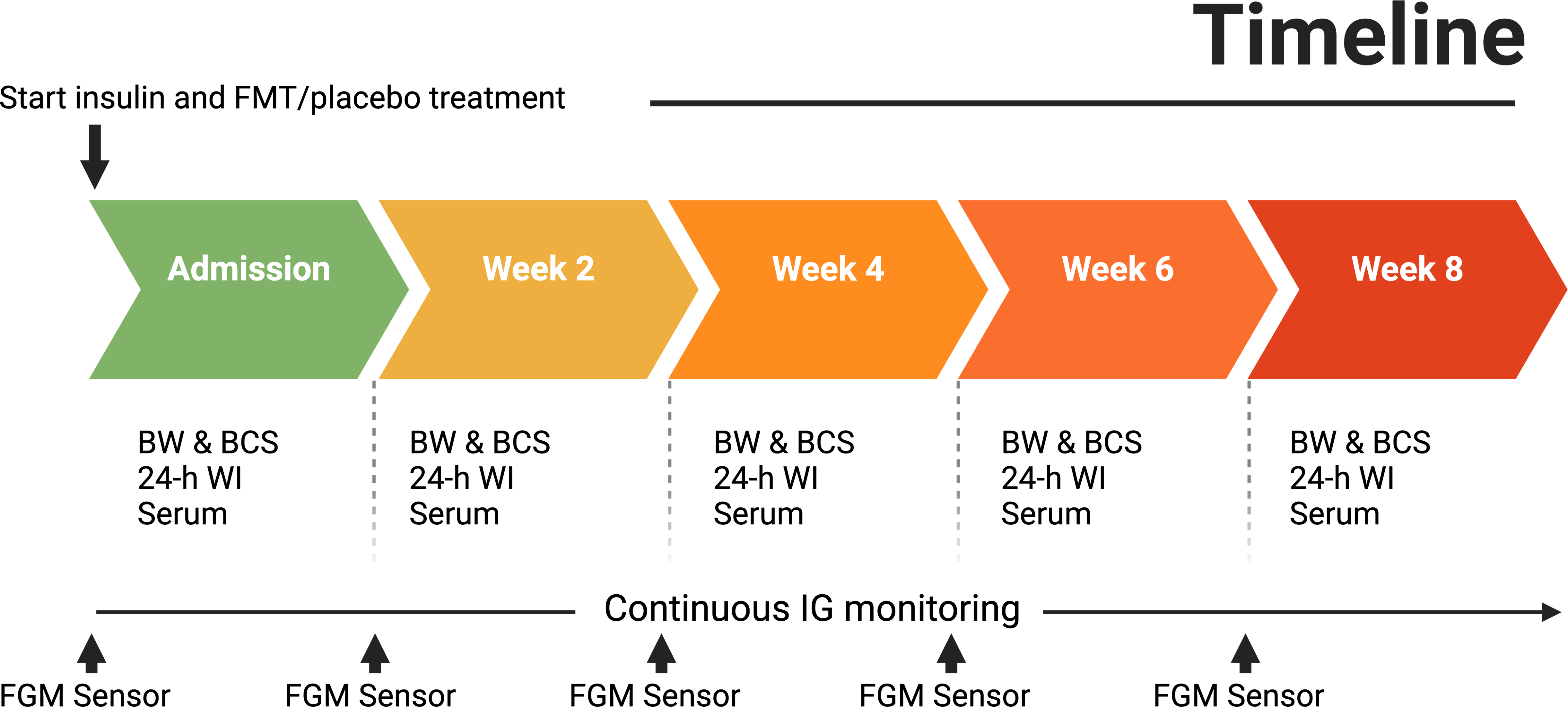


**Figure 1.** Study timeline and assessment schedule. Dogs were transitioned to Toujeo insulin and began treatment with LFP or placebo at admission. Evaluations included body weight (BW), body condition score (BCS), 24-hour water intake (WI), and serum collection at admission and biweekly through week 8. Flash glucose monitoring (FGM) sensors enabled continuous interstitial glucose (IG) monitoring throughout the study.

Throughout the study, dogs wore a previously validated flash glucose monitor (FGM) device (Corradini et al. 2016). Owners scanned sensors every 6 hours and uploaded daily glucose readings to a secure Libreview account (https://www.libreview.com).

**Insulin Treatment**

Upon enrollment, all dogs transitioned to Toujeo glargine insulin therapy, previously utilized in canine diabetes management (Tardo et al. 2024). Dogs weighing ≤15 kg received Toujeo SoloStar, whereas those >15 kg received Toujeo Max SoloStar, administered every 12 hours. Initial dosing was adjusted to match each dog's pre-enrollment dose. Owners followed a standardized sliding-scale dosing table, adjusting insulin based on postprandial interstitial glucose (IG) measured 60 minutes post-feeding (Fig. 2).


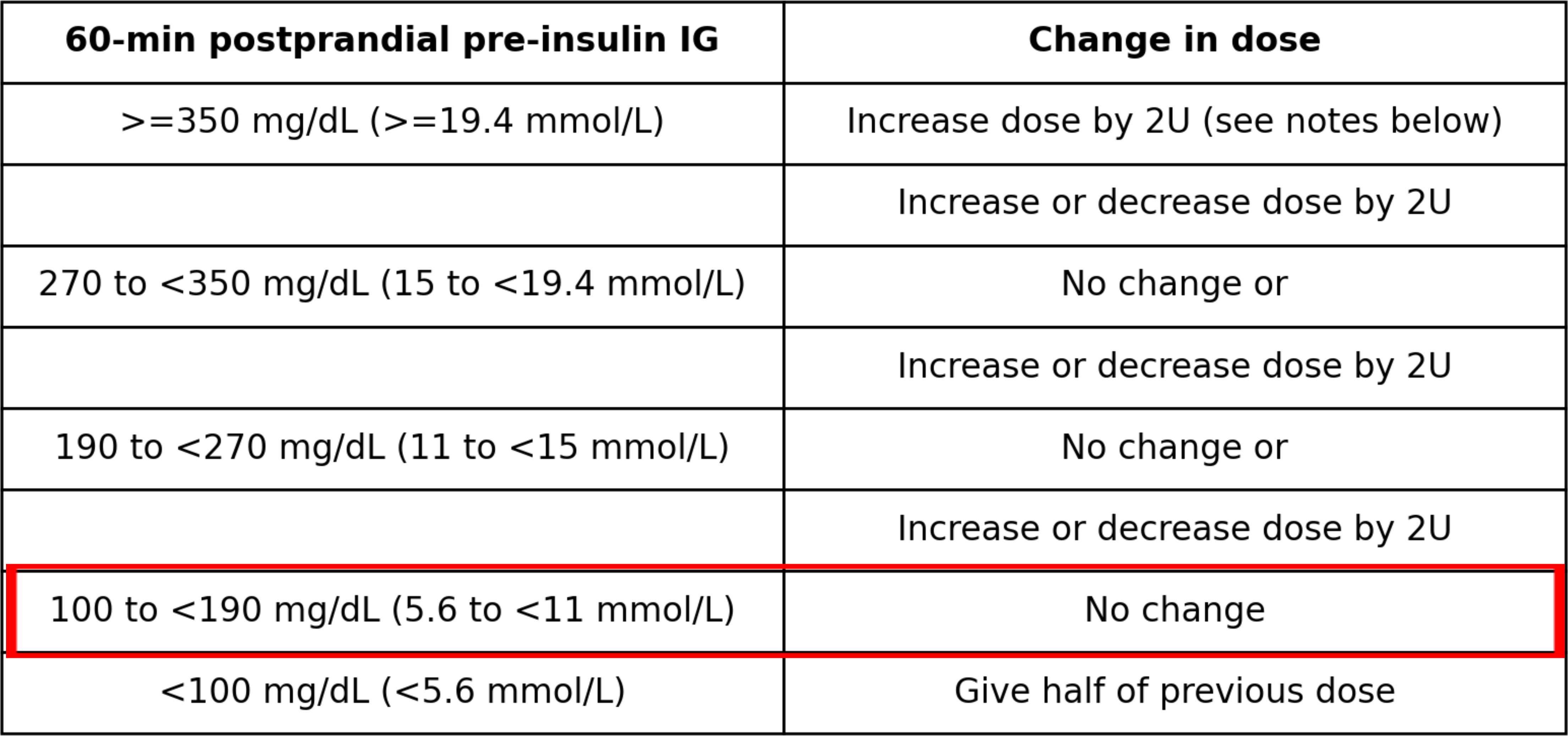


**Figure 2.** Sliding-scale insulin dosing protocol for diabetic dogs >15 kg (dogs ≤15 kg receive 1-unit adjustments between tiers). Insulin doses are adjusted based on 60-minute postprandial interstitial glucose (IG) levels. IG concentrations are categorized into five tiers with the target tier highlighted in red. Dose adjustments follow specific rules: reduction by half for below-target IG, 2-unit changes for tier transitions, and continuous increases for highest tier IG.

This strategy aimed to minimize dosing variability, reducing confounding effects and enhancing evaluation of LFP effects (Brown et al. 2025). Owners documented insulin administration daily in an online shared document.

**Treatment with lyophilized feces preparation**

Dogs in the LFP group were administered a daily dose of 1 g of lyophilized fecal material (ranging from 0.035 to 0.167 g/kg) encapsulated for oral delivery. This dosing regimen was adapted from a prior fecal transplantation protocol (Gal et al. 2021). Owners were asked to document each day’s capsule administration using an online shared document accessible to the principal investigator to ensure adherence. The lyophilized fecal capsules (size 0) were sourced from a commercial supplier (AnimalBiome INC., Oakland, CA), with donor dog demographics detailed in **Table 1**.

**Table 1.** Demographic descriptive statistics [mean ±SD; median (min, max)] of the dogs in the study.

|  | Donor | DM (Placebo + LFP) | Placebo | LFP |
| --- | --- | --- | --- | --- |
| Sex | 3 (CM), 1(SF) | 8 (CM), 3 (SF), 1(IF) | 4 (CM), 1 (SF), 1 (IF) | 4 (CM), 2 (SF) |
| Age (years) | 5.8 ± 0.5; 6.0 (5.0, 6.0)*^a^* | 10.3 ± 1.7; 10.1 (8.3, 13.2)*^b^* | 11.0 ± 1.9; 11.3 (8.3, 13.2) | 9.6 ± 1.2; 9.3 (8.4, 11.4) |
| Body weight (kg) | 27.6 ± 7.3; 27.8 (18.6, 36.4)*^a^* | 12.6 ± 7.8; 10.7 (5.2, 28.4)*^b^* | 9.1 ± 5.2; 7.1 (5.2, 19.0) | 16.1 ± 8.9; 13.0 (6.0, 28.4) |
| BCS (1–9 point scale) | 5 (4, 5) | 5 (4, 7) | 5 (4, 7) | 5 (4, 7) |
| Breed | Australian Cattle Dog, Border Collie/German Shepherd cross, Pitbull cross, German Shepherd | MBD (5), Pug (2), Chihuahua, Australian Cattle Dog, Poodle, Yorkshire Terrier, Maltese/Poodle cross | MBD, Chihuahua, Australian Cattle Dog, Poodle, Yorkshire Terrier, Maltese/Poodle cross | MBD (4), Pug (2) |

**CM**, castrated male; **SF**, spayed female; **IF**, intact female; **BCS**, body condition score; **LFP**, lyophilized feces preparation; **MBD**, mixed-breed dog; Different superscript letters represent significant differences between donor and diabetic dogs (p < 0.05).

Identically sized placebo capsules containing 1 g of cornstarch (range 0.053–0.192 g/kg) were also procured from the same vendor. Cornstarch at the administered dose is not recognized as a prebiotic for dogs according to current literature (Baioni et al. 2017). The randomization schedule and capsule assignments were managed exclusively by a veterinary pharmacist, who was responsible for distributing capsules at enrollment and remained uninvolved in other study activities to maintain double-blind conditions (**Fig. 3**).

**
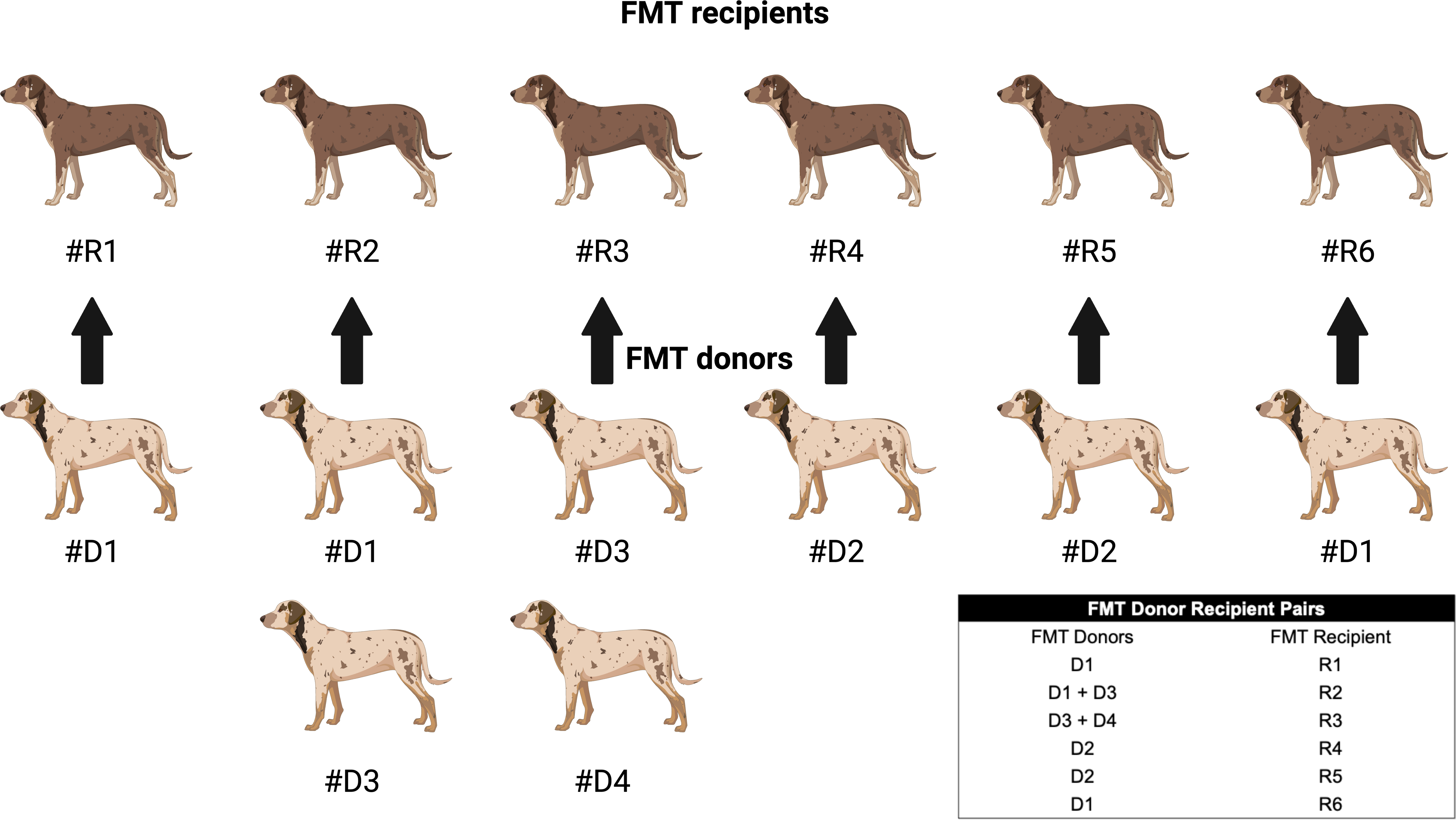
**

**Figure 3**. LFP Donor-recipient pairs.

**Untargeted Metabolomics**

Untargeted metabolomics was performed on serum samples by the Carver Metabolomics Core, University of Illinois Urbana-Champaign Roy. J. Carver Biotechnology Center. Samples were initially spiked with a mixture of deuterium and stable isotope labeled surrogate internal standards prior to processing, including TMAO-d9, L-alanine-d3, choline-d9, creatinine-d3, leucine-d10, lysine-d3, methionine-methyl-d3, glutamic acid-d5, L-carnitine-d9, L -phenylalanine-13C6, L-arginine-13C6, acetyl-carnitine-d3, tryptophan-d5, lauroyl-carnitine-d3, and D-glucose-13C6. Processed samples were dried and reconstituted with a mixture of instrument internal standards (50 µg/mL) and 5 µL 1-cyclohexyl ureido, 3-dodecanoic acid (CUDA) and injected onto the instrument for analysis. Samples were analyzed using a Dionex Ultimate 3000 series UHPLC system (Thermo Scientific) with a Q-Exactive MS system (Thermo Scientific), as described previously.(Bonini et al. 2020) Reversed-phase liquid chromatography (RPLC) was performed using a Waters Acquity ethylene-bridged hybrid (BEH) C18 column (100 mm × 2.1 mm; 1.7 μm) column maintained at 25 °C with a flow rate of 0.3 - 0.4 mL/min (Waters Corp). The mobile phases consisted of (A) water including 0.1% formic acid and solvent (B) acetonitrile including 0.1% formic acid. Spectra were acquired in positive and negative ionization mode.

Quality control throughout the LC-MS acquisition was instituted by randomization of the sequence, injection of instrument conditioning samples to equilibrate the LC–MS systems before study samples, inclusion of method blank analysis, monitoring of retention time, peak shape, intensity, and mass accuracy of internal standards, and injection of both pooled and external quality control samples throughout the sequence batch of samples.

All the LC-MS raw data files were performed using MS-DIAL ver.4.90 software for data collection, peak detection, alignment, adduct, and identification.(Tsugawa et al. 2015) The detailed parameter setting was as follows: MS1 tolerance, 0.005 Da; MS2 tolerance, 0.01 Da; minimum peak height, 100,000 amplitude; mass slice width, 0.05 Da; smoothing method, linear weighted moving average; smoothing level, 3 scans; minimum peak width, 5 scans. [M-H]-, [2M-H]- and [M+H]+, [2M+H]+ were included in adduct ion setting for negative and positive mode, respectively. Compounds were annotated by m/z and MS/MS spectra against an in-house library produced using chemical standards and the MassBank of North America and NIST20 libraries. The in-house library also enabled retention time match. Internal standards were monitored for retention time and intensity and PCA was used for multivariate statistics and visualization, specifically for outlier detection.

From the MS-DIAL output file, known metabolite identified features were filtered for isotopes, and subsequently features were removed if (sample max)/(blank average)<10 or pooled QC %RSD >30%. Features were evaluated for MSI level 1 matches.(Sumner et al. 2007) Next, positive and negative mode data were combined, and some replicate identifications removed by retaining features with MSI level 1 match. Remaining replicate features without MSI level 1 match were filtered by retaining those with the highest MS-DIAL total score. Manual spectra match confirmation was also executed. Following removal based on the previously mentioned sample max/blank average and QC %RSD, all unknown features not positively identified (unknown compounds) were retained. Sample peak heights (semi-quantitative) were normalized to the average internal standard response.

Known compounds with matched m/z, MS2, and retention time (using in-house chemical standards) were considered MSI level 1 (Sumner et al. 2007). Known compounds with strictly m/z and MS2 matches were considered MSI level 2. Unknown metabolites were considered MSI level 4.

Metabolite profiling was conducted by the Carver Metabolomics Core, University of Illinois Urbana-Champaign Roy J. Carver Biotechnology Center, as previously described (Southey et al. 2021). Each sample was analyzed using a gas chromatography–mass spectrometry (GC-MS) system consisting of an Agilent 7890 gas chromatograph (Agilent Technologies), Agilent 5975 MSD, and HP 7683B autosampler. GC was performed on a ZB- 5MS (60 m × 0.32 mm I.D. and 0.25 μm film thickness) capillary column (Phenomenex). The inlet and MS interface temperature was 250°C, and the ion source temperature was adjusted to 230°C. A 1μL was injected with a split ratio of 7:1. The helium carrier gas was held at a flow rate of 2mL/min. Isothermal heating was set at 70°C for 5 min, followed by an oven temperature increase of 5°C/min to 310°C, then the final 10min at 310°C. The MS was operated in a positive electron ionization (EI) mode at 69.9eV ionization energy with the scan range at m/z 30–800. Peaks were identified using the Automatic Mass Spectral Deconvolution and Identification System v2.71 (National Institute of Standards and Technology, MD) software and a custom-built MS database derived from in-house chemical standards and the National Institute of Standards and Technology database for annotation confirmation. All data were normalized to the internal standard (hentriacontanoic acid at 10 mg/mL).

**Sample Size Estimation**

Because metabolomic studies generate extensive and complex datasets, there are currently no universally accepted a priori methods for calculating sample size, especially for designs involving repeated measures or multiple experimental groups. Therefore, our sample size determination was guided by precedent in published studies that have reported biologically meaningful results using cohorts of 10 to 28 total dogs, typically with 6 to 12 dogs per treatment group and serum metabolomics as the primary endpoint (Horvatic et al. 2025; Lawrence et al. 2019; Minamoto et al. 2015; O'Kell et al. 2017; Rubic et al. 2023). To enhance the sensitivity and discriminatory power of our study, we aimed to enroll 24 dogs (12 per group for LFP and placebo).

**Statistical Analyses**

Statistical analyses utilized SAS Version 9.4 (SAS Institute Inc., Cary, NC) and R (Version 2023.06.2+561). Normality of demographic, interstitial glucose, and 24-hour water intake data were assessed via Q-Q plots, histograms, and Shapiro-Wilk tests. Descriptive statistics included mean (±SD) and median (min, max). The variable 24-hour water intake had a log-normal distribution after log transformation.

Linear mixed models analyzed interstitial glucose and log-transformed 24-hour water intake, accounting for repeated measures within subjects. Fixed effects included treatment group, week, and their interaction, with random effects for individual dogs. Pairwise comparisons were performed using Fisher’s Least Significant Difference, adjusted by Tukey's method. Ordinal BCS data, and non-normally distributed age and body weight were evaluated with NPAR1WAY.

Metabolomic data underwent missing-value filtering and imputation (features with >50% missingness were removed; remaining NAs were set to 0 and then replaced with 1/5 of the minimum non-zero value per metabolite), followed by median-scaling (per metabolite) and natural-log transformation. Unsupervised analyses included principal component analysis (PCA) and hierarchical clustering. Linear mixed-effects models tested metabolite abundance differences, with fixed effects for phenotype (placebo vs FMT), time, and their interaction, and a random intercept for subject. To control false discovery across metabolites, omnibus (ANOVA) p-values from the mixed-effects models were converted to q-values using the qvalue approach; metabolites with q < 0.2 in the global model were considered candidates for downstream inference. Pairwise post-hoc contrasts were then performed using estimated marginal means (emmeans); in the code shown, contrasts were computed for phenotype (placebo vs LFP). Metabolite set enrichment analysis (MSEA) tested whether specific metabolic sub-pathways were overrepresented among metabolites differing between groups. Metabolites were ranked using a composite statistic defined as (−log(q-value from the phenotype contrast)) × (log2 fold change), producing a signed list in which large positive ranks indicate higher relative abundance and large negative ranks indicate lower relative abundance in the placebo group versus LFP group. Enrichment was then evaluated with the fgsea R package (v1.22.0), which returned normalized enrichment scores (NES) for each sub-pathway. Benjamini-Hochberg adjustments controlled false discovery rates (q-values < 0.2 were considered significant).

Lipid indices were derived from GC–MS intensities: SCD1_16 = 16:1/16:0, SCD1_18 = 18:1/18:0, DNL = 16:0/18:2, and DNL_alt = (16:0+16:1)/18:2. Long-chain acylcarnitines (palmitoyl-carnitine, C16; stearoyl-carnitine, C18) were obtained from LC–MS. All ratios and acylcarnitines were natural-log transformed (offset 0.001) to assume a log-normal distribution. Linear mixed-effects models (PROC MIXED) were fitted with fixed effects for treatment (LFP vs placebo), time, and their interaction, modeling within-dog correlation using AR(1) repeated measures (subject = dog). Least-squares means were estimated for the main effects and sliced by time.

**References**

Baioni E, Scanziani E, Vincenti MC, Leschiera M, Bozzetta E, Pezzolato M, et al. (2017) Estimating canine cancer incidence: findings from a population-based tumour registry in northwestern Italy. BMC Vet Res 13: 203 doi:10.1186/s12917-017-1126-0

Bonini P, Kind T, Tsugawa H, Barupal DK, Fiehn O (2020) Retip: Retention Time Prediction for Compound Annotation in Untargeted Metabolomics. Anal Chem 92: 7515-7522 doi:10.1021/acs.analchem.9b05765

Brown R, Barko P, Ruiz Romero JDJ, Williams DA, Gochenauer A, Nguyen-Edquilang J, et al. (2025) The effect of lyophilised oral faecal microbial transplantation on functional outcomes in dogs with diabetes mellitus. J Small Anim Pract doi:10.1111/jsap.13865

Corradini S, Pilosio B, Dondi F, Linari G, Testa S, Brugnoli F, et al. (2016) Accuracy of a Flash Glucose Monitoring System in Diabetic Dogs. J Vet Intern Med 30: 983-988 doi:10.1111/jvim.14355

Gal A, Barko PC, Biggs PJ, Gedye KR, Midwinter AC, Williams DA, et al. (2021) One dog's waste is another dog's wealth: A pilot study of fecal microbiota transplantation in dogs with acute hemorrhagic diarrhea syndrome. PLoS One 16: e0250344 doi:10.1371/journal.pone.0250344

Horvatic A, Kules J, Gelemanovic A, Smolec O, Pirkic B, Pecin M, et al. (2025) Combining Metabolomics and Proteomics to Reveal Key Serum Compounds Related to Canine Intervertebral Disc Herniation. Metabolites 15 doi:10.3390/metabo15060396

Lawrence YA, Bishop MA, Honneffer JB, Cook AK, Rodrigues-Hoffmann A, Steiner JM, et al. (2019) Untargeted metabolomic profiling of serum from dogs with chronic hepatic disease. J Vet Intern Med 33: 1344-1352 doi:10.1111/jvim.15479

Minamoto Y, Otoni CC, Steelman SM, Buyukleblebici O, Steiner JM, Jergens AE, et al. (2015) Alteration of the fecal microbiota and serum metabolite profiles in dogs with idiopathic inflammatory bowel disease. Gut Microbes 6: 33-47 doi:10.1080/19490976.2014.997612

O'Kell AL, Garrett TJ, Wasserfall C, Atkinson MA (2017) Untargeted metabolomic analysis in naturally occurring canine diabetes mellitus identifies similarities to human Type 1 Diabetes. Sci Rep 7: 9467 doi:10.1038/s41598-017-09908-5

Rubic I, Weidt S, Burchmore R, Kovacevic A, Kules J, Eckersall PD, et al. (2023) Metabolome Profiling in the Plasma of Dogs with Idiopathic Dilated Cardiomyopathy: A Multiplatform Mass-Spectrometry-Based Approach. Int J Mol Sci 24 doi:10.3390/ijms242015182

Southey BR, Bolt CR, Rymut HE, Keever MR, Ulanov AV, Li Z, et al. (2021) Impact of Weaning and Maternal Immune Activation on the Metabolism of Pigs. Front Mol Biosci 8: 660764 doi:10.3389/fmolb.2021.660764

Sumner LW, Amberg A, Barrett D, Beale MH, Beger R, Daykin CA, et al. (2007) Proposed minimum reporting standards for chemical analysis Chemical Analysis Working Group (CAWG) Metabolomics Standards Initiative (MSI). Metabolomics 3: 211-221 doi:10.1007/s11306-007-0082-2

Tardo AM, Fleeman LM, Fracassi F, Berg AS, Guarino AL, Gilor C (2024) A dose titration protocol for once-daily insulin glargine 300 U/mL for the treatment of diabetes mellitus in dogs. J Vet Intern Med 38: 2120-2128 doi:10.1111/jvim.17106

Tsugawa H, Cajka T, Kind T, Ma Y, Higgins B, Ikeda K, et al. (2015) MS-DIAL: data-independent MS/MS deconvolution for comprehensive metabolome analysis. Nat Methods 12: 523-526 doi:10.1038/nmeth.3393
